# A Multimodal Dataset to Investigate Task-Evoked Negative BOLD Response and Neurodegeneration

**DOI:** 10.1038/s41597-026-07081-x

**Published:** 2026-03-25

**Authors:** Bardiya Ghaderi Yazdi, Sindy Ozoria, Seyed Hani Hojjati, Peter Chernek, Jenseric Calimag, Xiuyuan Hugh Wang, Qolamreza R Razlighi

**Affiliations:** 1 Department of Radiology, Weill Cornell Medicine Brain Health Imaging Institute, Quantitative Neuroimaging Laboratory, New York, NY USA; 2 Department of Radiology, Weill Cornell Medicine, Brain Health Imaging Institute, New York, NY USA

**Keywords:** Cognitive ageing, Alzheimer's disease, Protein aggregation, Human behaviour, Neural ageing

## Abstract

The Quantitative Neuroimaging Laboratory Dataset provides magnetic resonance imaging (MRI) modalities and two resting-state and twelve task-based functional MRI (fMRI) tapping into four cognitive domains (episodic memory, fluid reasoning, processing speed, and crystallized memory). It also includes three positron emission tomography (PET) scans ([18 F]Fluorodeoxyglucose (FDG), Florbetaben, and MK-6240), plus neuropsychological assessments, and vital signs. Currently, 356 participants consented (97 young: 20 ~ 40 years; and 259 elderly: 60 ~ 80 years), while 259 completed at least one scan. We uploaded 4688 MRI/fMRI and 719 PET scans (232 Florbetaben, 251 FDG, and 236 MK-6240). 189 participants completed all scan modalities. All imaging underwent an in-house, pre-processing pipeline developed for each modality. This dataset aims to characterize the spatial and temporal properties of the brain’s hemodynamic response in the opposite direction (i.e., brain deactivation), its task dependency, and its interaction with the brain’s large-scale functional connectivity networks. Ultimately, this will enable the translation of neuroimaging findings into personalized medicine approaches that better characterize and predict individual pathologies in neuropsychiatric diseases.

## Background & Summary

Functional magnetic resonance imaging (fMRI) has revolutionized our understanding of the brain function through its networks and their interactions during task and rest^1^. Since fMRI’s inception, researchers have been puzzled over an intriguing observation that many brain regions exhibited hemodynamic response in an opposing direction during various tasks. This observation was not exclusive to fMRI and has also been reported in positron emission tomography (PET) imaging studies. In fact, the discovery of the brain’s Default Mode Network (DMN) was made possible by examining the hemodynamic response in the opposite direction in multiple PET and fMRI studies^2,3^. Despite these genuine breakthroughs, the brain’s hemodynamic response in the opposite direction, hereafter referred to as negative blood oxygenation level-dependent (BOLD) response (NBR), is poorly understood and is often overshadowed by its counterpart, the positive BOLD response (PBR).

This dataset aims to provide the necessary neuroimaging and cognitive data to enhance our understanding of the brain’s NBR and to utilize NBR to elucidate brain networks’ individualized functioning, interactions during tasks and rest, and dysfunction in the context of aging and neurodegenerative diseases, which can then be applied to personalized medicine. Furthermore, disruptions to the NBR are inextricably linked to neuropathology in cognitively unimpaired (CU) participants^4–6^ and patients with mild cognitive impairment (MCI) or Alzheimer’s disease (AD)^6,7^, Therefore, to clearly understand brain function and health, it is imperative to delve into the characteristics and underlying mechanisms of the NBR in healthy and clinical populations^6,8^.

The Quantitative Neuroimaging Laboratory Dataset (QNLD) provides a multimodal neuroimaging dataset of young and elderly participants during both resting state and diverse task-based conditions. QNLD contains cross-sectional neuroimaging data mainly focusing on tb-fMRI, which evaluates four cognitive domains (fluid reasoning, episodic memory, processing speed, and crystallized memory), each assessed with three similar cognitive tasks^9,10^. These tasks have been modified to be executable inside the MRI scanner. In addition to tb-fMRI data, this dataset also includes resting-state functional connectivity (FC), magnetization-prepared rapid gradient echo (MPRAGE), fluid-attenuated inversion recovery (FLAIR), susceptibility weighted imaging (SWI), quantitative susceptibility mapping (QSM), diffusion MRI (dMRI), pseudo-continuous arterial spin labeling (pCASL), and data from three PET imaging modalities, which are often used in aging and neurodegenerative studies ([18 F]Fluorodeoxyglucose (FDG), Amyloid-beta (Aβ), and Tau). The QNLD dataset is not limited to neuroimaging data; it also holds neuropsychological assessment data of diverse scientifically approved cognitive tasks evaluating distinct cognitive domains. To our knowledge, such a dataset has not been published before, and the existing studies on task-evoked fMRI often neglect the NBR.

All participants were recruited through random market mailing and screened for dementia, handedness, health, psychiatric conditions, contraindications for MRI, poorly controlled medical conditions, and neuropsychiatric disorders. Participants were excluded if these factors were present. This ongoing project will include longitudinal data on participants with pathological patterns of Aβ or tau every 2.5 years and healthy, normal participants every five years. The scans will be repeated in the Brain Health Imaging Institute (BHII) of the Radiology Department at Weill Cornell Medicine (WCM). This project will repeat MK-6240 and Florbetaben (FBB) PET scan modalities, resting state-fMRI (rs-fMRI) scans, structural MRI scans, and four task-based fMRI (tb-fMRI) scans (one for each cognitive domain) for future longitudinal data.

The dataset presented here has already been utilized in several previous manuscripts. More research is ongoing. In one study, our dataset was used as a healthy control group^11^. In another study, Aβ increases in inter-network FC were shown to facilitate the early stage of tau accumulation^12^. An independent component analysis (ICA) study showed that different tau accumulation patterns in CU individuals are associated with differential cognitive decline^13,14^. Other studies showed that network-based Aβ pathology^15^ and a differential pattern of tau deposition^16^ can predict subsequent cognitive decline in CU, and that regional Aβ and tau proteins have a remote (non-overlapping), inter-regional association^17^ mediated by between‐network FC^18–20^ and are stage-dependent^21^.

One study using this dataset showed the FC of the entorhinal cortex predicted tau in CU^22^. Other studies investigated the relationships between tau and Aβ on cortical thickness^23,24^. This dataset was used to develop and evaluate the tau progression index (TPI), an individualized predictor of Alzheimer’s Disease trajectory based on subject-specific connectomes^25^. Further, this dataset was used to investigate the interaction between NBR and FC^26^ as well as the linearity of the NBR and distinct HRFs for each DMN sub-region^27^. Also, this dataset showed that large within-network FC remained relatively intact throughout normal aging and pre-clinical AD^28,29^ while the NBR changed with aging and preclinical AD for tasks improving with age. Finally, this dataset showed that a higher magnitude of NBR was related to better performance in cognitive domains, both improving and deteriorating with age and that NBR surpassed PBR in task specificity^30^. This dataset aims to provide all the necessary neuroimaging and cognitive data to facilitate the investigation and understanding of the NBR and its framework. Understanding normal NBR will inform researchers how the brain networks function individually, interact during tasks and rest, and help reveal the mechanisms of malfunctioning during pathological aging, neurodegenerative, and psychiatric diseases.

## Methods

### Recruitment

Participants within a ten-mile radius of the Citigroup Biomedical Imaging Center at Weill Cornell Medicine (WCM) were recruited by random market mailing services from Aplus Services, Inc., which utilizes census and New York City Department of Motor Vehicles data to obtain population information and demographics. Further recruitment was facilitated by posting leaflets around different college campuses and receiving referrals from enrolled individuals^9^.

### Screening and consenting

Interested participants were invited for a 20-minute phone screening. Inclusion criteria included (1): ages 20–40 or 60–80 (2), normal cognition (3), English-speaking (at least grade 5 proficiency) (4), right-handed, and (5) no contraindications to MRI and PET scans. Cognitive normality was determined during screening by asking whether participants had any significant memory challenge impacting their personal or professional life, or if they had ever been diagnosed with a memory-affective disorder. English proficiency was assessed based on conversational fluidity and whether the participant attended an English-speaking primary school. Participants were classified as right-handed if they scored 50 or higher on the Edinburgh Handedness Inventory^31^. Participants must have been willing to undergo the MRI scans, PET scans, and neuropsychological assessment.

Exclusion from the study was enforced when the health history implicated either the participant’s safety or the study’s integrity. Participants were excluded based on the following criteria: (1) presence of non-MRI compatible metal implants such as a pacemaker, (2) presence of excessive metal near the brain which may induce artifacts, (3) excessive, recent substance abuse, (4) usage of certain medications such as benzodiazepines, antipsychotics, mood stabilizers, and insulin injections, (5) history of head trauma or neurological and psychiatric conditions, (6) existence of uncontrolled health conditions such as high cholesterol or high blood pressure. A separate consent call was scheduled to review the study’s goal, explain each visit, and outline the benefits and risks associated with participation.

**Fig. 1:**
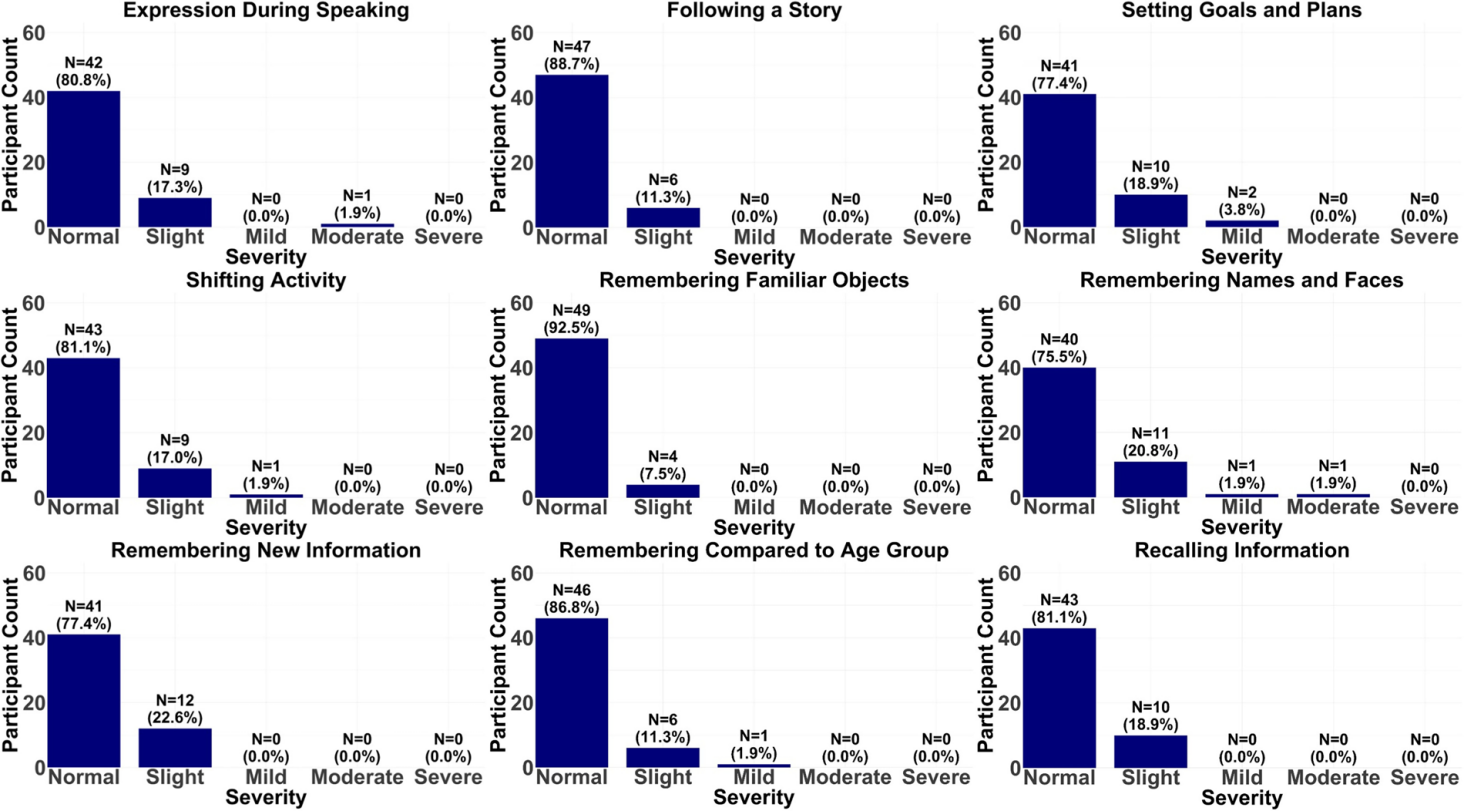
Cognitive Change Index survey distributions for the young group. The following descriptions explain the meaning of each index and what the severity or degree of impairment entails for both Figs. 1, 2. Each index corresponds to the self-perception of their ability. 1. Expression During Speaking. 2. Following a Story. 3. Setting Goals and Plans. 4. Shifting Activity. 5. Remembering Familiar Objects means that upon placing a familiar object somewhere, how well they think they can remember where they put the object. 6. Remembering Names and Faces describes their ability to remember a new person’s name and/or face after meeting someone for the first time. 7. Remembering New Information is the self-perception of their ability to recall information after hearing or being told new information. 8. Remembering Compared to Age Group is the self-perception of their own cognition compared to others of the same age group. 9. Recalling Information is the ability to recall information actively instead of passively in their own opinion. Each participant rated their impairment in performance within each Cognitive Change Index using one of five different severity criteria: 1. Normal ability: No change or better than five years ago. They don’t report problems in daily activities and need no assistance. 2. Slight/Occasional problem: Minimal or subtle change from five years ago, occurring less than once a week. They might feel slightly slower or less efficient, but they don’t need assistance with their ability. They are probably not noticeable to most other people. 3. Mild problem: Some changes from five years ago. They feel a bit worse and may perform related activities more slowly or less efficiently. They occasionally may need a little help. Their condition may be noticeable to others. 4. Moderate problem: Clearly noticeable change from five years ago. They report frequent problems that may affect daily complex activities or occur several times per week. They often need assistance in performing complex activities. 5. Severe problem: Much worse than five years ago. They have continuous problems and are unable to independently perform complex activities, requiring ongoing assistance or supervision.

**Fig. 2:**
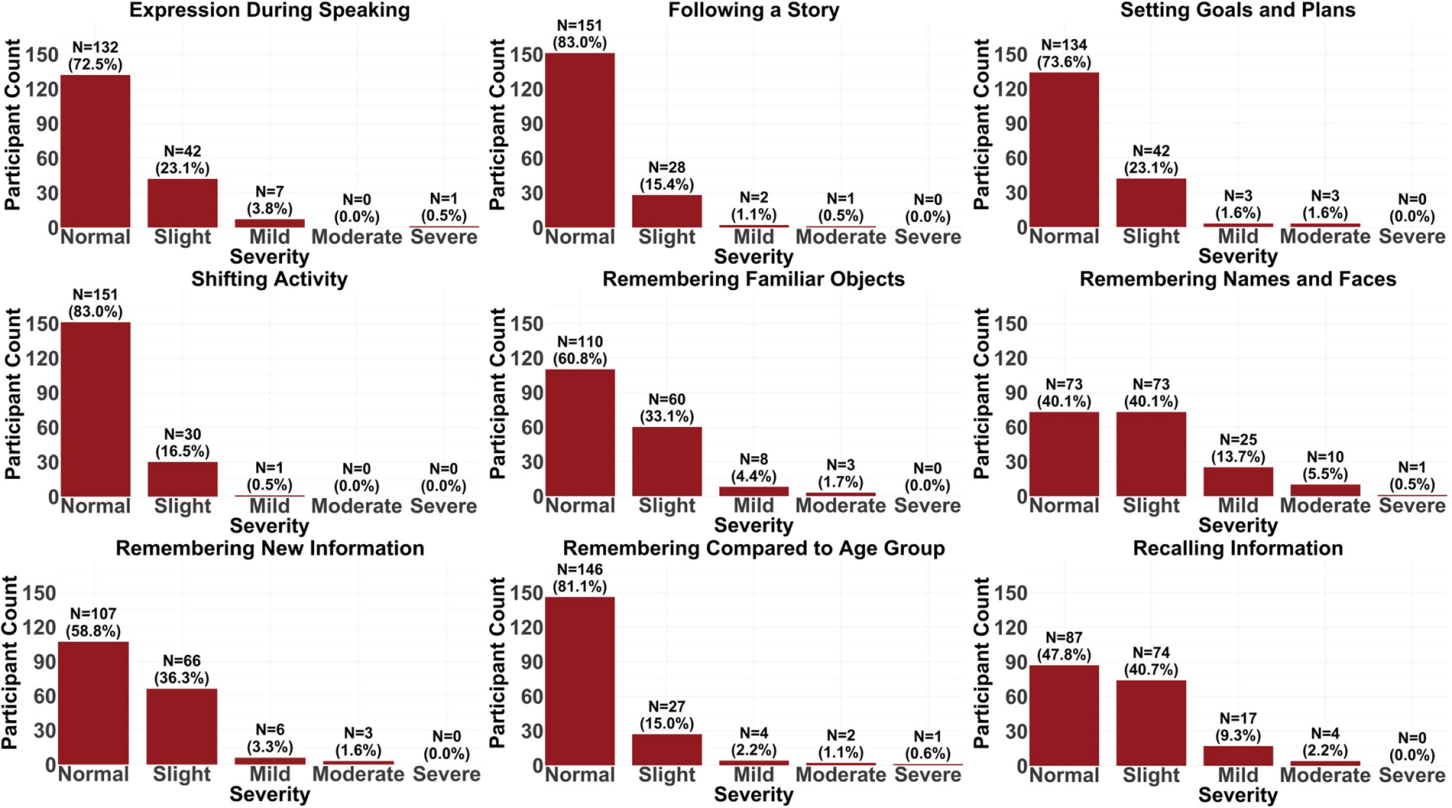
Cognitive Change Index survey results for the elderly group.

An emphasis was placed on the elderly group’s screening to ensure that they did not meet the criteria for dementia or MCI. The Montreal Cognitive Assessment (MoCA) and the Cognitive Change Index Questionnaire were used for identifying impaired participants. In the Cognitive Change Index Questionnaire, participants completed a self-rating scale that specifically asked about their current skills, problem areas, daily functioning, activities, and the severity of any current problems, ranging from normal ability to severe problems. Bar plots from nine sample questions assessing a wide array of cognitive areas during aging can be seen for the young (Fig. 1) and the elderly groups (Fig. 2). In addition to the above screening criteria, a neuroradiologist reviewed the T1 scan after the first visit to check for any clinically significant findings. Any significant findings were conveyed to the research team and the participant’s primary care physician. If these findings posed any issue for our study, the participants were also excluded.

More comprehensive surveys were completed through self-administered forms, including the Beck Depression Inventory^32^, Epworth Sleepiness Scale^33^, Pittsburgh Sleep Quality Index^34^, a simplified version of the Menstrual Health Questionnaire available in the PhenX toolkit Protocol 101101^35^, Family History created using the patient family history guidelines published by the American Medical Association, and Periodontal Health Questions of the Oral Health Questionnaire^36^.

### Ethics statement

After addressing any questions and concerns, participants signed the e-consent form online from Research Electronic Data Capture (REDCap)^37^ and were enrolled in the study. An in-person, paper consent form was also obtained during each participant’s first visit. Our study procedure was certified by the WCM Institutional Review Board (IRB) under IRB# 19-11021058. In addition to consenting to all study procedures, consent for data sharing and storage was also obtained. Within the consent form, there is a statement that specifically informs participants of the creation of a repository for their imaging data, which will be shared with other researchers in the field. Should they not agree to this portion of the study, participants were excluded from participation.

### Procedure

All the imaging and behavioral data were collected during three separate visits to the WCM campus. The scanning began in October 2020, and the last session was completed in June 2024. In total, twelve tb-fMRI were administered for each participant. Of these scans, three were episodic memory, three processing speed, three crystallized memory, and three fluid reasoning tasks in addition to two rs-fMRI scans, one with ASL, and another with FDG PET. The fMRI tasks were divided into two sessions, each composed of six fMRI tasks and one PET scan. Before each MRI session, a 45–60-minute pre-scan training session was done with each participant. The order of tb-fMRI scan acquisition was randomized and counter-balanced annually to ensure no bias was present in the order in which fMRI tasks were performed across sessions. Each MRI session was usually combined with one PET scan. The FDG PET scan was often paired with the first session, and the MK-6240 PET scan with the second session. Finally, the third session combined the FBB PET scan with cognitive testing. The aim was to finish all three sessions in three weeks. Currently, 356 participants consented (97 young and 259 elderly). The demographic overview is presented in Table 1. Of the 356 consented, 259 completed at least one scan from which we uploaded 4688 MRI/fMRI and 719 PET scans (232 Florbetaben, 251 FDG, and 236 MK-6240), in which 225 participants completed all three visits. Some people attended 3 visits but missed some modalities.

**Table 1:** Demographics of participants with at least one complete scan.

Cohort Characteristics	Young (n = 62)	Elderly (n = 197)
Age range mean ± SD	20–40 years 27.8 ± 5.3	60–80 years 68.6 ± 5.7
Education (years), mean ± SD	16.5 ± 2.0	17.0 ± 2.4
Sex (M/F)	32/30	100/97
Self-reported race (may select multiple)		
White	31	167
African American	7	15
Asian American/Pacific Islander	12	8
Hispanic	9	2
Other	6	11

A comprehensive neuropsychological battery of tasks was administered to all participants to putatively assess the following eight cognitive domains: 1) dementia screen and general intellectual functioning, 2) learning and memory, 3) processing speed, 4) executive functions, 5) fluid reasoning, 6) vocabulary, 7) visuo-construction, and 8) sensory-motor speed. The first session lasted approximately four to five hours to complete. Participants first underwent a 90-minute FDG PET session, after which they were given a short break. The actual within-scanner time for FDG PET was approximately 30 minutes. The additional 60 minutes were allocated to the participant’s preparation before and after the scan (i.e., pre-scan prep: glucose testing pre-injection, acquisition of vitals, intravenous (IV) catheter insertion, and FDG bolus injection; post-scan: acquisition of vitals). Participants were given a lunch break and trained outside the scanner in six out of twelve fMRI tasks until they were comfortable performing each task. In addition to the fMRI scans, each participant was scanned for typical structural brain imaging (T1-weighted, T2-weighted, T2*-weighted, and Diffusion-weighted), which could take up to 20 minutes. During fMRI scans, participants completed six of the twelve cognitive tasks they encountered during training, each lasting ten minutes. tb-fMRI scans and non-task scans (structural and resting-state) were alternated, ensuring that participants were not continuously answering questions. Participants were instructed to answer the questions to the best of their ability and to remain as still as possible. The total scan time did not typically exceed 120 minutes. A sample scan sequence for the first MRI session is as follows (tb-fMRI, MPRAGE-T1, tb-fMRI, FLAIR, tb-fMRI, T2, tb-fMRI, pCASL, tb-fMRI, rs-fMRI, tb-fMRI).

The second session also lasted approximately four to five hours. During this session, participants underwent fMRI/MRI scans, an MK-6240 PET scan, and a blood draw. As in the first session, participation in the second session began with an MK-6240 PET scan that lasted two hours, including preparation, tracer uptake, and scan time. Once completed, participants underwent 45–60 minutes of training in the fMRI tasks they would later complete inside the scanner. In addition to the remaining six tb-fMRI scans, two resting state scans (one with fMRI and one with pCASL) were added. A sample scan sequence for the second MRI session is as follows (tb-fMRI, rs-fMRI, tb-fMRI, dMRI one, tb-fMRI, dMRI two, tb-fMRI, QSM, tb-fMRI, MRA, tb-fMRI). The fMRI/MRI acquisition order between the two visits could vary from participant to participant due to excessive motion, technical difficulty, change in the task paradigm, participant discomfort, and counter-balancing for future statistical analysis.

During the third visit, all participants received a 2.5-hour comprehensive set of cognitive assessments and a two-hour FBB PET scan, including preparation, FBB uptake, and scan time. Although unlikely, any fMRI/MRI scans not obtained in the first two visits could be performed on a separate visit.

Study data were collected and managed using REDCap electronic data capture tools hosted at WCM^38,39^. REDCap is a secure, web-based software platform designed to support data capture for research studies, providing 1) an intuitive interface for validated data capture; 2) audit trails for tracking data manipulation and export procedures; 3) automated export procedures for seamless data downloads to common statistical packages; and 4) procedures for data integration and interoperability with external sources.

### Blood sampling, processing, and storage

A blood sample of 50 milliliters was taken from each participant, usually during the participant’s second visit. The blood sample was drawn using the same IV catheter for MK-6240 PET scanning. Blood samples were collected in 10 mL BD Vacutainer Lavender top ethylenediaminetetraacetic acid tubes and 5 mL BD Vacutainer gold top Serum Separator tubes. Once collected, the blood-filled tubes were centrifuged, and then each blood product–plasma, serum, and buffy coats were aliquoted into pre-labelled 0.5 mL self-standing cryovials and stored within subject-specific cryoboxes in the BHII blood repository. Cryovials containing buffy coat samples were sent for genetic Apolipoprotein E epsilon-4 allele (ApoE4) analysis to the Clinical and Translational Science (CTSC) core lab. Specimens were processed at the CTSC core lab in WCM or by a trained research team member at the BHII. All samples were labeled with anonymized IDs to ensure they were unidentifiable by unauthorized personnel, and they were stored in refrigerators with a temperature range of −70 °C to −90 °C. Samples were processed under the site’s laboratory safety policies. Appropriate personal protective equipment was required. The APOE analysis results provided data for carriers, noncarriers, and unavailable status. We are currently in the process of genotyping our participants by applying a Genome-Wide Association Study as part of the New York Genome Sequencing Project.

### MRI acquisition parameters

The same research dedicated 3.0 T Siemens Magnetom Prisma scanner with a 64-channel head-coil and an 80 mT/m gradient system was used to acquire all MRI/fMRI scans in QNLD. It has been shown that MRI data acquired from multiple scanners often cause significant heterogeneity in the data, which will be alleviated in this project by using a single scanner^40^.

First, every individual underwent a scout localizer to determine the position and set the field of view and orientation. Next, a high-resolution T1-weighted MPRAGE structural scan was collected [TR/TE = 2400/3 ms; FA = 9°; FOV = 256 × 256 mm; matrix-size = 512 × 512; voxel-size = 0.5 mm × 0.5 mm × 0.5 mm; 320 axial slices] for each participant to enable localization and spatial normalization of the functional data. All fMRI data are acquired with a multiband T_2_* weighted echo-planar imaging (EPI) pulse sequence [repetition time (TR)/echo time (TE) = 1008/37 ms; flip angle (FA) = 52°; Field of View (FOV) = 208 × 208 mm; matrix-size = 104 × 104; voxel-size = 2 mm × 2 mm × 2 mm; 72 axial slices; multiband factor (MB) = six]. Each fMRI scan lasted for ten minutes (600 volumes). Scans in between tasks lasted between 5–10 minutes.

Two dMRI scans [TR/TE = 3230/89.20 ms; flip angle = 78°; FOV = 21 × 21 cm; matrix size = 140 × 140; voxel size = 1.5 mm × 1.5 mm × 1.5 mm, 92 axial slices; two b-values = 0, and 3000 $\frac{s}{m{m}^{2}}$, and MB = six] (five-minute, 37-seconds each) with 98 directions were acquired with opposite phase encoding directions.

The pCASL data were acquired [TR/TE = 4000/33.8 ms, flip angle = 120°, FOV = 240 × 240 mm, base resolution = 96 and phase resolution = 59% (≈ 96 × 57), 60 transversal slices (slice thickness = 2.5 mm; distance factor = 50%), interleaved ascending acquisition, bolus duration = 700 ms, and inversion time = 1800 ms] while suppression mode was GRAY-WHITE, and 11 measurements were collected (magnitude reconstruction).

A 3D multi-echo gradient echo sequence with flow compensation was used to acquire QSM data [TE1 ~10 = 6.10, 10.12, 14.14, 42.28 ms; TR = 50 ms; FA = 15, matrix size = 256 × 256; FOV = 208 × 256 mm, slice thickness = 1 mm; and 144 axial slices]. Its acquisition period was four minutes and 29 seconds. Post-reconstruction using the Morphology Enabled Dipole Inversion method was used to reconstruct QSM and SWI images from these multi-echo scans^41,42^. FLAIR and SWI scans were acquired with Alzheimer’s Disease Neuroimaging Initiative 2 acquisition parameters.

Each session included six tb-fMRI scans. The phase encoding direction was alternated between consecutive tasks and used retrospectively for geometric distortion correction. In the case of any visual impairments, participants were instructed to use contact lenses, if possible; otherwise, they were provided with MRI-safe visual aids. The total MRI scan time per session was between 1.5–2 hours.

### PET imaging acquisition

All PET scans were acquired on a similar Siemens Biograph mCT–S in dynamic and 3D imaging mode. Each PET scan was acquired in a separate session at least one week apart. Vital signs were obtained before and after each PET scan and recorded in the QNL dataset.

#### FDG PET acquisition

To assess the spatial distribution of resting cerebral metabolic rate of glucose, participants underwent FDG PET. Participants were instructed to fast for at least four hours before their scan time. Our staff checked the blood sugar level of each participant via a finger stick blood test upon arrival for their scan. In cases of high blood sugar at or above 200 mg/dL (11 mmol/L), the participants’ scans were rescheduled to a later date. They received an IV injection of 5 ± 0.5 mCi (185 MBq) of the radiotracer. Next, participants were instructed to rest quietly in a room for 30 minutes without distractions such as reading or phone usage, allowing tracer uptake. Brain images were acquired in successive 5-minute frames over 30 minutes.

#### FBB PET imaging

As an AD biomarker, the presence of Aβ deposition was evaluated in each participant using the ^*18*^*F-Florbetaben tracer*. First, an IV catheterization was performed. Next, participants were injected with a slow single IV bolus at 60 seconds or less of 8.1 mCi ±20% (300 MBq) tracer. The scan started 90 minutes after tracer injection. A low-dose computed tomography (CT) scan was acquired for attenuation correction of the PET data. Brain images for each PET scan were acquired in 4 × 5-minute frames over 20 minutes.

#### MK-6240 PET imaging

For imaging tau accumulation in the brain, every participant received a slow, single IV bolus of 185 MBq (5 mCi) ±20% (maximum volume 10 mL) of MK-6240 tracer before imaging at 60 seconds or less. Two different methods were used to acquire the PET images. The first method, “*coffee break protocol*”, involved splitting the image acquisition into two parts. This method began imaging immediately after injecting the bolus and continued for 60 minutes. The participant was then removed from the scanner, given a 30-minute break, and imaging resumed for 30 minutes. This created the two-time points of 0–60 minutes and 90–120 minutes post-injection. The second protocol/image acquisition had only a single time point, which began at 90–120 minutes post-injection. This protocol was performed as six five-minute frames for a 30-minute PET acquisition. A total of 120 participants underwent the “*coffee break protocol*” while the remainder underwent the second protocol.

### PET scan visual reading

Two neuroradiologists reviewed the images for tau and Aβ deposition separately. The neuroradiologists were each provided with deidentified images. The images were then visually interpreted, and participants’ scans were classified as positive or negative for both Tau and Aβ at a global, whole-brain scale, and lobar regional level. Lobar characterization for each Aβ scan involved the evaluation of tracer uptake in the frontal, cingulate/precuneus, parietal, occipital, and temporal lobes. For tau, regional characterization was noted for the frontal, parietal, occipital, and lateral temporal lobes. Tau deposition was also evaluated separately in the medial temporal lobe. Moreover, tau deposition was further characterized by hemisphere symmetry and spread. Visual readings were performed using Functional Magnetic Resonance Imaging of the Brain (FMRIB) Software Library (FSL) by overlaying the PET scans over the participant’s T1-weighted MPRAGE scan after rigid-body registration. Freesurfer regional borders were also available in case the neuroradiologist wanted to examine the exact location of different region borders.

### Pre-MRI preparation and training

Before each MRI/fMRI scan, participants were escorted to a designated study room within the imaging center. The room was free of visual and auditory distractions. A Microsoft Surface laptop equipped with a USB-connected right-handed Celeritas button-press device (https://pstnet.com/products/celeritas/) was provided. Participants were allowed to examine and familiarize themselves with the hand device and its wrist straps before training began.

**Fig. 3:**
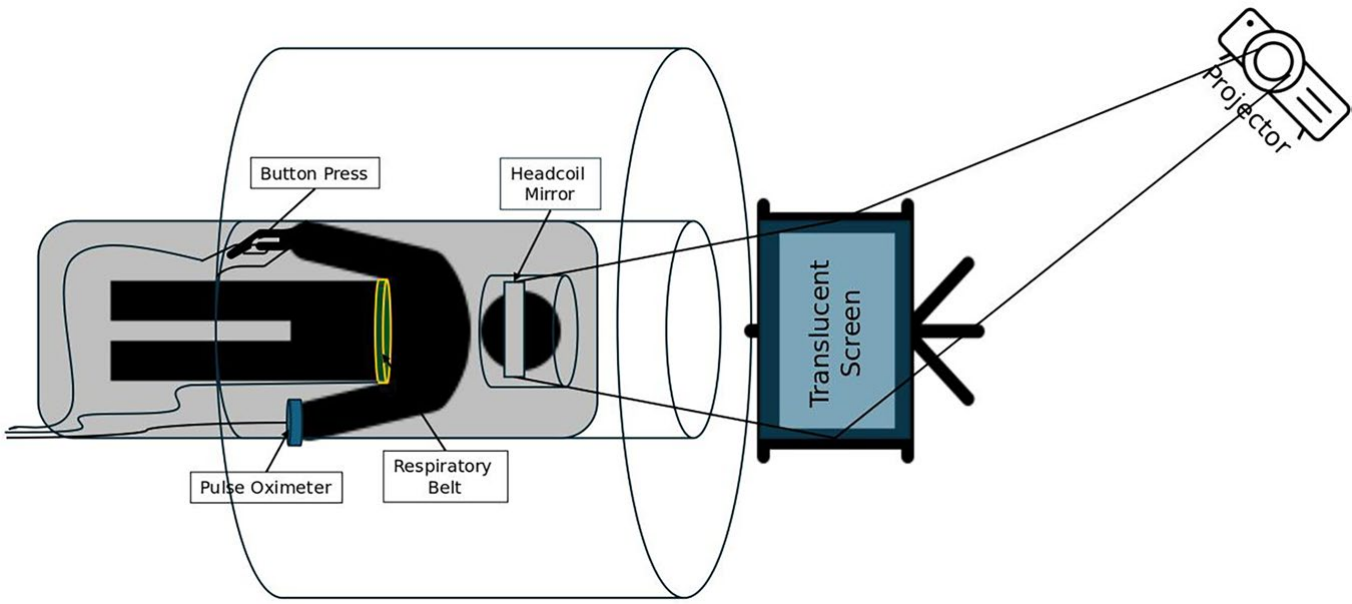
A sample of fMRI task administration for a task-based fMRI. This shows the participant lying inside the scanner while viewing the screen, which shows ongoing trials. Meanwhile, they have to choose between 5 options using a hand device.

Once comfortable, the study staff explained the imaging procedures and how each task would be completed during scanning. Participants were informed that while lying in a supine position, a head coil with an angled mirror would be placed over their head. They were also informed that the task would be projected onto the translucent screen located at the far end of the scanner bore, and they would be able to see the task in the angled mirror fixed on the head-coil. (Fig. 3)^43^ Questions regarding comfort and the scanning procedure were addressed before training began.

### Pre-MRI training protocol

Training protocols were also developed in E-Prime. Each task’s training sequence consisted of a small set of 5–7 sample questions per task. Despite the reduced number of questions, the structure and timing closely mirrored the in-scanner versions. Each training set began with a task title screen, followed by an instructional page describing the task and displaying a hand diagram indicating response options and finger placement. Participants then viewed a “GET READY” prompt and a fixation screen containing a central plus sign before the task began. A key difference between the training and in-scanner tasks was the inclusion of immediate feedback during training. Participants were informed whether each response was correct and were allowed to select an additional choice until the correct response was selected.

Each task’s training sequence began with two untimed questions. During which, participants were reminded that, during scanning, all questions would be timed and should be answered as quickly and accurately as possible. For the first untimed question, a study team member provided step-by-step guidance to ensure comprehension of the task structure and response format. The second question was completed independently. Study personnel observed the responses and provided clarification if multiple incorrect attempts suggested a misunderstanding of the task.

After the two untimed items, all remaining practice questions followed the same timing parameters as their corresponding within-scanner tasks, with the only difference being that received feedback such as: “It was correct. Well done!”, It was incorrect. “Please try again”, or “You have to answer in 35 seconds”, reinforcing the importance of accuracy and timeliness. Pre-MRI training took 45–60 minutes, depending on the participant’s ability in learning and answering the practice questions.

### Quantitative and qualitative measures of task familiarity

Upon completion of each training module, participants’ performance accuracy (percent correct) and the number of response attempts were automatically recorded by E-Prime. Participants were required to achieve a minimum of 80% accuracy across practice items before proceeding to the MRI session. Participants who scored below this threshold or required additional clarification repeated the training until they demonstrated adequate task comprehension. Immediately following training, participants were asked about their comfort and familiarity using a 5-point scale. These ratings were used to confirm participant readiness and pre-scan preparedness. Together, these objective performance metrics and subjective ratings ensured that all participants entered the MRI session with demonstrated and quantifiable understanding of each task, thereby minimizing in-scanner learning effects and reducing between-subject variability.

### fMRI experimental setting

All fMRI experiments were designed with E-Prime 3 (https://pstnet.com/products/e-prime/), which employed an event-related fMRI task paradigm to effectively investigate both the magnitude and dynamics of the hemodynamic response function for the NBR. Each task was implemented in a multiple-choice format for ease of response within the scanner. The number of answer choices available varied according to the specific task. Before the start of each task, similar to the training phase, a screen was prompted with instructions, and the assigned numbers relative to their finger placement were displayed (Fig. 3). For both the training and fMRI scanning sessions, the following finger placement corresponded to each answer: thumb with choice number one, index finger with choice two, middle finger with choice three, ring finger with choice four, and pinky finger with choice five (Fig. 3). Due to the differences in task difficulty, the number of trials could not be balanced across tasks. Instead, the task/rest time ratio was counterbalanced across all tasks in a way that the result of dividing the average time participants were engaged in task trials by the mean rest time (not engaged in trials) was equivalent across all tasks. The total duration of each fMRI task was set to ten minutes to control for the variability in BOLD response as well as FC networks due to the total duration of the fMRI run.

### Stimulus presentation

**Fig. 4:**
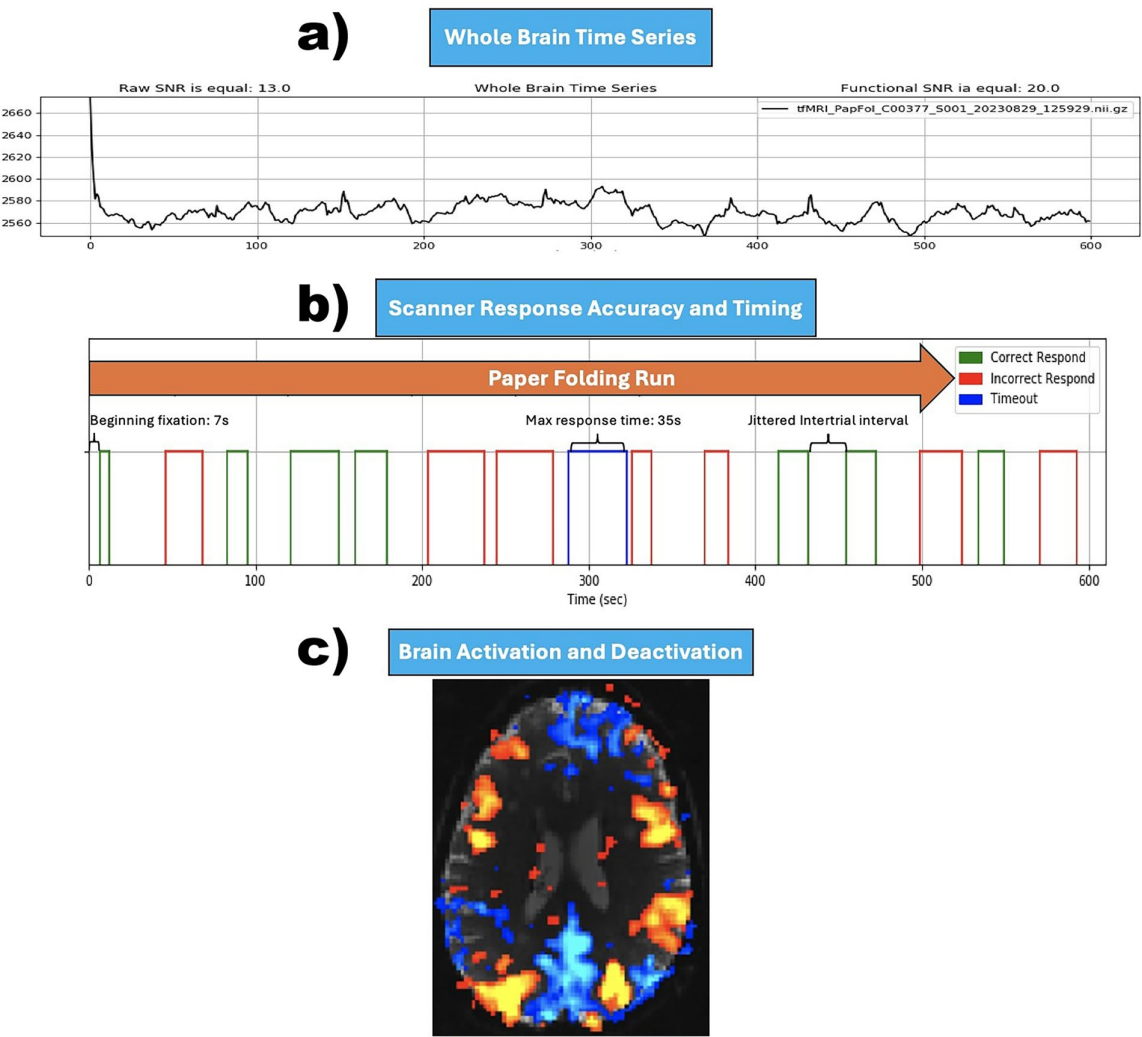
(**a**) A typical whole brain mean fMRI time series, (**b**) A sample box-car plot representing the fluid reasoning (paper folding) task trials timing. As is seen, the inter-trial intervals are jittered. Green squares show correct responses, and red ones correspond to incorrect responses. If the participants did not respond within the maximum allowable time, the trial will be marked as a timeout shown in blue. (**c**) A typical pattern of the brain activation and deactivation from a single participant.

All tasks were designed visually and presented within a square/rectangle aligned to the horizontal meridian and projected to a translucent screen placed at the far end of the scanner’s bore. Each participant was supine within the magnet bore, wearing noise-isolating MR-safe earbuds, and viewed the screen using a mirror placed on the head coil. Tasks were projected on the translucent screen via an LCD projector located at the corner of the MRI room. Participants watched the tasks on the screen via a mirror system in the head coil. Participants responded to questions via a LUMItouch response system (Photon Control Company). (Fig. 3) The E-Prime software-controlled task administration collected the response (to quantify accuracy) and response time (RT) on a computer within the MRI control room. Task onset and the start of the fMRI acquisition were electronically synchronized. (Fig. 4b).

### fMRI tasks

The fMRI experiments were designed to measure brain activation/deactivation patterns during twelve different cognitive tasks, specifically designed to tap into four different cognitive domains (three tasks for each cognitive domain) and two resting-state scans. The four cognitive domains were fluid reasoning, processing speed, episodic memory, and crystallized memory. The three fluid reasoning tasks were Paper Folding, Matrix Reasoning, and Letter Sets; the three processing speed tasks were Digit Symbol, Letter Comparison, and Pattern Comparison; the three episodic memory tasks were Logical Memory, Word Memorization, and Paired Associate; and the three crystallized memory tasks were Antonym, Synonym, and Picture Vocabulary. All trial timings were jittered only once for each task, making every participant experiencing the same exact jittered trial timing but unable to anticipate the onset of the trials. The primary behavioral variables for all tasks were RT and ACC (accuracy). Performance was recorded for all trials within the scanner. Below is a brief explanation of each fMRI task design categorized by cognitive domain.

### Fluid reasoning

#### Paper Folding

During this task, participants were presented with two, three, or four folding patterns of a blank square sheet one at a time from left to right on the top of the screen and a hole was punched through the folded paper on the last fold. The participants were asked to choose from one of the five unfolded sheets below, which contained the correct hole arrangements using the button-press device. Each scan started with a 7-second beginning fixation (BF) cross and was followed by the first stimulus. If the participant exceeded the maximum allowed length of trial (LoT) for any trial, a time-out was recorded for that question. The timing parameters for this task were as follows: BF = 7 seconds; trials per run (TpR) = 15; LoT = 35 seconds; inter-trial interval (ITI) was drawn from a random uniform distribution in the range of U[17.91, 26.91] seconds. The timings can be seen in (Fig. 4b). A pre-training session consisted of five practice problems, where feedback was provided for each practice, and the correct answer was explained to the participant, if requested. If the participant was having difficulty, a video-guided solution would be shown through the surface computer, or if needed, a research coordinator would guide the participant with an actual paper and a hole puncher as a visual enactment of the sample question within the same room^44^.

#### Matrix Reasoning

This task was adapted from the Raven Matrix Reasoning^45^. During this task, participants identified a specific pattern from a figure series shown in a 3 × 3 matrix. The matrix consisted of nine cells, with the bottom-right cell missing. Participants were presented with five possible figures and selected one that best completed the missing, bottom-right cell. For Matrix Reasoning, the timing parameters were the same as Paper Folding task: BF = 7 seconds; TpR = 15; LoT = 35 seconds; ITI = U[15.27,24.27] seconds. Training for this task was structured similarly to the paper-folding task, without the use of videos or hands-on instruction.

#### Letter Sets

Participants were presented with five sets of letters. Each set had four letters printed on a different line. The five lines are organized vertically from the top of the screen. Four sets had a standard rule (i.e., ascending letters, same number of vowels, etc), while one set did not follow that rule. Participants were instructed to select the letter sequences that did not follow the common rule among other choices or did not share the same pattern as the other choices. Item structure and training procedures mirrored those of the other fluid reasoning tasks. For Letter Sets, the timing parameters were as follows: BF = 7 seconds; TpR = 17; LoT = 30 seconds; ITI = U[14.46, 24.46] seconds^44^. Training for this task was structured similarly to the paper-folding task, without the use of videos or hands-on instruction.

### Processing speed

#### Digit Symbol

One table was shown at the top of the screen during this task. The table consisted of codes of numbers on the first row (one through nine randomized); each paired to an associated symbol on the second row. An individual pair of “digit symbols” was presented below the table. Participants were required to indicate whether the probe pair matched one of the digit symbol pairs from the code table above the screen by using buttons one or two on the hand device. For all three perceptual speed tasks, button one was used if the pairs matched, and button two was used if they did not match. Participants were instructed to respond as quickly and accurately as possible during this task (0~5 seconds RT). The task started with an eight-second BF, and the other timing parameters were as follows TpR = 60; LoT = 5 seconds; ITI = U [2.2, 12.2] seconds. Pre-training of the Digit Symbol task included five practice trials^10^.

#### Letter Comparison

Participants observed two strings of three to six letters next to each other and decided if the strings were identical or different by pressing one of two buttons on the hand device. The Letter Comparison task was initiated with BF = 7 seconds and the following timing parameters: TpR = 74, LoT = 4 seconds, and ITI = U[1.78, 11.78] seconds. The structure of pre-training was similar to the Digit Symbol task, except there were ten practice problems instead of five^46^.

#### Pattern Comparison

In this task, two abstract figures were presented, each with a varying number of lines and connected at different angles. Participants were asked to determine if the figures were identical or different from each other by pressing button one or two on the hand device. The timing parameters were as follows: BF = 7 seconds; TpR = 74; LoT = 4 seconds; ITI = U [1.93, 11.934] seconds. Ten practice problems were given during pre-training^46^.

### Episodic memory

#### Logical Memory

In this task, participants read three different short stories projected on the screen and answer several questions about each story. Each story is broken into smaller segments and presented for 30 seconds. Participants were instructed to pay close attention to the details and remember all specific information given in each story inside the scanner. Each story was followed by a 10-second retention period, where a blank page with a fixation cross was projected on the screen. Next, participants responded to ten detailed four-choice questions about each story by pressing one of the four corresponding buttons. Each four-choice question was projected on a single page, and participants had a maximum of 10 seconds to respond to each question. The next story was shown to the participants after 15 seconds waiting period, and the structure and timing of the second and third story was similar to the first. The timing of the questions in each story was jittered, but the maximum and minimum jittered ITIs were the same. The timing parameters were BF = 7 seconds, TpR = 30; LoT = 10 seconds; ITI = U [5.84, 14.84] seconds. Pre-training involved reading one story, followed by 10 probe questions^10^.

#### Word Memorization

In this task, participants were presented with five lists of words, each list containing 15 words. The words on each list were projected one at a time on the screen for 1.5 seconds, followed by a fixation cross of 0.5 seconds. Ten seconds after the last word in each list, participants were presented with four words and were asked, “which word existed in the list?”. Six questions with jittered ITI were asked, in which they had to select from four answer choices and respond by pressing the corresponding button on the hand device. There was a 15-second wait time between the last question and the first word of the subsequent list. The first letter of each answer choice was similar to the correct word from the list to ensure that the participants encoded the entire word into memory. The timing parameters were BF = 7 seconds, ITI = U[3.14, 18.14] seconds. During pre-training, one 15-word list was presented to each participant, followed by five problems^47^.

#### Paired Associates

In this task, participants viewed six unrelated word pairs presented on a screen one at a time, each for four seconds, where each pair was followed by a fixation cross for two seconds. Participants were instructed to remember each word pair. Nine seconds after all the pairs were shown, a six-consecutive-probe word with four possible word choices below appeared at the top of the screen for six seconds. Participants were instructed to select the word initially paired with the probe word as quickly and accurately as possible. There was a 15-second lapse time between the last question of a list and the first pair of the subsequent list. In total, five sets of six paired words with follow-up questions with similar structure and timing were presented. The timing parameters for this task were as follows: BF = 7 seconds; TpR = 30; LoT = 6 seconds; ITI = U[2.36, 17.36] seconds. Pre-training consisted of one list of six-word pairs and five probe questions^10^.

### Crystallized memory

#### Synonym

In this task, participants were asked to match a given word with the choice with the closest meaning. An uppercase probe word was presented at the top of the screen inside a square with four numbered choices. Participants were instructed to respond quickly and accurately by pressing one of four buttons corresponding to the matching synonym. This task consisted of 40 trials with the following timing parameters: BF = 7 seconds; LoT = 10 seconds; ITI = U[4.75, 14.75] seconds. Pre-training consisted of one set of five synonym questions^46^.

#### Antonym

In this task, participants were asked to match a given word with the choice with the most opposite meaning. The structure and timing of the task was identical to the Synonym task. The timing parameters for Antonym were as follows: BF = 7 seconds; TpR = 40; LoT = 10 seconds; ITI = U[4.26, 14.26] seconds. There were ten pre-training questions as opposed to five in Synonym^46^.

#### Picture Vocabulary

This task was adapted from the NIH toolbox. During this task, four images, each placed on one of four corners of the screen, were displayed along with a single word probe in the center of the screen. The objective was to view each of the four images and select the one that best matched the meaning of the word in the center of the screen. This task consisted of 40 trials over 10 minutes. The timing parameters for this task were BF = 7 seconds, TpR = 40; LoT = 10 seconds; ITI = U[5.66, 14.66] seconds. During pre-training, ten questions were given to each participant to allow enough time to understand the task concept^48^.

#### Neuropsychological assessment

An extensive battery of neuropsychological assessments was administered to evaluate the cognitive abilities of study participants. This assessment incorporated elements from the National Alzheimer’s Coordinating Center (NACC) Uniform Dataset (UDS) for the T-cog Neuropsychological Battery, UDS v3.0, March 2015^49^, alongside several computer-based tasks administered using a Microsoft Surface laptop with Inquisit software referred to here as Millisecond Tasks. Each Millisecond Task was administered utilizing a stylus pen and a mouse provided by the research coordinator and adhered to the procedures outlined in the Inquisit user manual. A comprehensive performance report for each task was generated. The NACC battery was administered both verbally and with paper and pencil. The paper and pencil tasks were scored by two different research assistants in order to ensure agreement. A third research assistant would only be needed for scoring in the unlikely case where the first two scorers could not resolve a discrepancy in the grading. The final score recorded was the one that all evaluators agreed upon. All behavioral data were written into a logfile under the “file-display” directory of the dataset (https://openneuro.org/datasets/ds006148/versions/1.0.5/file-display/QNLD_logfile.csv), which was supplied with the BIDS neuroimaging data. Below are the details of the Neuropsychological tasks administered in this study.

### Computer-based tasks

#### Symbol Search

Participants were provided a Microsoft Surface computer, where they were first introduced to the Millisecond Symbol Search task. This included clear instructions and three practice problems that they must complete before proceeding. After the practice, participants were presented with the actual task consisting of 10 rows of symbols. Each row contains seven symbols, with two on a gray background (on the left) and five on a white background (on the right). Participants are instructed to search for a matching symbol from the gray background (left side) among the five symbols on each row’s white background (right side). If a match is found, they must click on the corresponding symbol. If no match is found, participants select the “NO” button at the end of the row. This process continues for two minutes, with ten additional rows presented after the initial ten are completed. A circle around the chosen symbol indicates each selection. The number of correct responses within the two-minute window determines the participant’s performance on the task^50^.

#### Four-Choice Reaction Time

This task was completed using the Millisecond. In this task, which lasts approximately 2.5 minutes, participants are presented with four horizontally arranged black boxes on the screen. After a fixed amount of time, one of the boxes turns red. Participants are asked to select the red box as fast as possible using either the mouse or their finger^51^.

#### Tower of London

The Tower of London task was initially developed by psychologist Tim Shallice. Our study used an electronic version available via the Millisecond. The premise of this task is to use the given three different pegs and three different colored balls to recreate a displayed pattern with the minimum number of moves. The caveat to this task is that the number of moves for each pattern displayed is restricted so that only one ball may be moved at a time, and the number of attempts to answer the question correctly is also restricted. Although the number of attempts allowed for each pattern is consistently three throughout the task, the number of allowed moves varies from question to question. If the participant fails to recreate the displayed pattern after three attempts, the question is skipped, and the participant is moved on to the next problem. In the same way, participants may also elect to skip the question and move on to the next by selecting the next button on the screen. The Tower of London lasted for ten minutes from start to finish. The setup includes one practice trial (problem with two moves), and the test consists of twelve test trials (two problems with two moves, two problems with three moves, four problems with four moves, four problems with five moves), tested sequentially in a fixed order^52^.

#### Baddeley’s Grammatical Reasoning

This task was first developed in 1968 by Alan Baddeley. In this task, participants are presented with several statements about how the letters A and B are presented and related. The participant is then asked to judge whether the statement is true or false relative to the AB-paired order presented. An example of a statement presented is “A follows B” paired with the two letters “BA”. Here, participants are asked to judge whether the letter pair correctly represents the statement by clicking “true” or “false.” The test times out after three minutes. The test lasts four minutes, with a one-minute setup^53^.

#### Shipley and Number Series

This is a ten-minute task comprised of word and number series patterns. Participants are given ten minutes to answer 24 fill-in-the-blank questions in this task. In the number series portion of this task^54^, participants are given five number series questions within a specified time frame (default: 4.5 minutes) with five-choice options to select their answer. Once the possible choice is determined, the participant selects the response that best matches their desired answer from the multiple-choice options. In the Shipley portion of this task, which is a Millisecond version of the task described by Walter C. Shipley^55^, participants are given various words or letters in a specific pattern. Like the number series portion, the participant is asked to find a pattern with the displayed letter arrangement and select an option that best completes the pattern.

#### Manikin test of spatial orientation and transformation

We used the Millisecond version of this task, first described by Benson and Gedye^56^. It is a 7-minute task that tests spatial orientation. In this task, a manikin is displayed. It has been superimposed onto either a red square or a green circle. The manikin itself shows several simple features that can help orient the position of the manikin in relation to the participant, such as buttons, slit pockets, a belt buckle when facing towards the participant, hair on the head, and back pockets when it is facing away from the participant.

Each manikin holds a red square in one hand and a green circle in the other. The orientation of these manikins varies. In each trial, the manikin’s position might be upside down or right side up, can face towards the participant (‘forward’), or face away from the participant (‘backward’). The participant is asked to decide if the manikin holds a shape congruent with the background (e.g., the red square) in its left or right hand. They should press the left or right buttons correspondingly as quickly and accurately as possible. There were 16 trials for this task.

#### Trail Making Test

Traditionally, the Trail Making Test (TMT) is composed of two parts using paper, a pen, and a timer. Our administration of this task utilizes the Millisecond on a Microsoft Surface computer and a stylus pen. Like its paper-based counterpart, participants are instructed to use the stylus pen and connect the circled numbers sequentially. In the paper-based task, as the participants make an error, coordinators point it out instantly for the participant to correct. In this version, a mistake or missed numbered circle will prevent the participant from continuing the task, and the correct path will be highlighted in yellow. 25 circles are displayed on a white screen in both parts. The circles in Part A are numbered from one to 25. Here, the participants are instructed to make a trail between the circles using the stylus pen in numerical order as fast as possible. In Part B, 25 circles are once again presented. However, in this instance, the circles combine numbers from one to 13, and the letters span from A to L. Here again, the participant is required to draw a line connecting each circle but alternating between numbers and letters during the test. The number of seconds taken to complete each part decides the score. Total run time, including setup, is five minutes. The scoring requires converting raw scores, which are in seconds, to a ten-point scale^57^.

### Paper-based and verbally administered tasks

#### Design Fluency

This task is part of the Delis-Kaplan Executive Function System (DKEFS). In the Design Fluency task, participants are assessed on their problem-solving initiation, ability to generate visual patterns fluently, creativity in drawing new designs, and capacity for simultaneous processing while adhering to task rules and inhibiting previously drawn responses. The task involves a sheet of paper containing 35 squares, each filled with a variable number and arrangement of dots, which differ across conditions. Despite the variation in dot patterns, all three conditions follow the same basic rules. Participants must create a shape using only four straight lines, where the lines must begin and end at a dot. Lines may connect through other dots and may also intersect.

In the first condition, participants are given a sheet with only black dots and are tasked with completing as many designs as possible within 60 seconds. In the second condition, the sheet contains black and white dots, but participants are instructed to use only the white dots to create their designs within a 60-second time frame. The third condition presents participants with a sheet containing a mix of five black and five white dots per square, requiring participants to alternate between black and white dots while creating each design.

Before each condition begins, participants receive a brief practice session during which the rules are explained; they familiarize themselves with the task, and any necessary corrections are made before the timed trial^58,59^.

#### The Montreal Cognitive Assessment

The Monteral Cognitive Assessment (MoCA) administered in this study was the modified Blind MoCA Version 8.1/Telephone^60^. This version is a condensed version of the full MoCA assessment, lasting approximately ten minutes, and is scored out of 22. MoCA guidance states that an additional point can be given to individuals with twelve or fewer years of education. An addition of six points would make it more comparable to the full MoCA in terms of scores. However, we elected to leave the scoring out of 22 to keep it solely based on performance since our participants all had twelve or more years of education. This Blind MoCA included a short immediate recall where the participants heard a list of five words and were asked to repeat them back to the coordinator, regardless of the order. This was done twice, and after the second time, the participant was reminded to remember the five words for future recall. The participant was then given a list of five numbers and asked to repeat the numbers back in the exact order stated. Once complete, a set of three numbers was read to the participant, and they were asked to repeat the numbers in reverse order based on how they heard them. Afterwards, a sequence of 29 letters was read to the participants. While listening to the letter sequence, participants were instructed to tap the table or their shoulders whenever they heard the letter A. This was followed by a test requiring the participant to count backwards by seven, starting at 100, until they reached the number 65. Then, two language sections were completed. In the first portion, the coordinator read two sentences to the participant that were asked to be repeated back verbatim. In the second portion, the participant was told the letter “F” and asked to state as many words that begin with the letter. These words must have adhered to the following rules: 1) no proper nouns, 2) no numbers, and 3) no added suffixes to the same word. The Blind MoCA was completed with three final tasks: abstract thinking, delayed recall, and orientation. The abstract thinking required the coordinator to read two groups of words and request that the participant state the relationship between the two groups. The delayed recall required the participant to repeat the initial five words stated to them at the beginning of the exam. The final task required the participants to confirm their orientation (i.e., date, month, year, place, and city). Each portion of the MoCA was awarded specific points that resulted in 22 points in total.

#### The Rey Auditory Verbal Learning Test

The Rey Auditory Verbal Learning Test (R-AVLT) is commonly used as a neuropsychological measurement for learning and verbal memory. During this task, participants were instructed to remember a list of 15 semantically unrelated words (list A) and recall as many words as possible during each of the trials. The entire trial (encoding/learning and recalling) with the same list of words (list A) was repeated five times. Next, a single learning session of 15 new words in list B, which the participant was asked to recall only once. After the recall of the list B word, the participant was asked to immediately recall all the words from list A only. 20–30 minutes later, a delayed free recall of the original list A items was obtained. This was followed by a recognition trial in which thirty words were presented verbally by the research assistant. The 30 words included list A words and 15 foils. Participants decided if the words belonged to list A or not by stating yes or no. The following tasks were completed during the 20–30-minute interval before beginning the delayed R-AVLT: Number Span Forward, Number Span Backwards, Four-Choice Reaction Time, and TMT. The Number Span Forward and Backward tasks were administered verbally. However, the remaining tasks, TMT and Four-Choice Reaction, were administered using the Millisecond on a Microsoft Surface computer^61^.

#### Craft story 21

The immediate Craft story 21 (CS-21) and delayed CS-21 evaluate verbal memory by assessing the ability to encode and recall a short story of a boy who loses a soccer ball. Participants listen to a paragraph and are instructed to repeat it as best as possible during immediate CS-21. The coordinators told the participants that they would have to remember the story later. After 20 minutes, individuals recalled story details as best as they could during delayed CS-21. Each recalled detail was scored as either verbatim or paraphrased^62^.

#### Number Span Test (Forward, Backwards)

The Number Span Test or Digit Span Test (Forward, Backwards) was developed to test the participant’s working memory by focusing on their ability to recall a series of numbers. In the Number Span Forward task (NSF), participants hear a series of spoken numbers and are then asked to repeat those numbers to the examiner in the exact order given. The length of the number series is repeated twice and then elongated. The longest number in the series is eight digits. Like its sister test, Number Span Backwards (NSB), the examiner reads a series of numbers to the participant; however, instead of repeating the numbers in the stated order, participants are asked to repeat the numbers in reverse order. In addition to measuring working memory, NSB also tests manipulation and each participant’s cognitive control level. With both NSB and NSF, the examiner continues to read each series until the participant erroneously repeats the reading series two consecutive times or until they reach the maximum number of series^63^.

#### Benson Complex Figure Copy

During this task, participants are instructed to copy a complex figure from an example with eight elements. The performance is assessed based on accuracy and placement, and participants can receive a maximum score of two per element. They receive one bonus point and score 17 when all elements are perfectly placed in proper proportions with clean connections and no extra lines. This task lasted four minutes. Benson Complex Delay was administered 10–15 minutes after the Benson Complex Copy, and four minutes were allowed for completion^64^.

#### Category Fluency

The Category Fluency task evaluates semantic verbal fluency. It requires participants to generate as many spoken words belonging to a given category as they can within 60 seconds. Scoring is based on the total number of unique words produced, the number of errors, and the number of correct words^65^. In this study, two fluency categories were used: animals and vegetables.

#### Verbal Fluency

In this task, participants were asked to state as many words as they could that began with the letters “F” and “L” in under 60 seconds. Participants were informed that words were not allowed to be proper nouns, numbers, or exact words with differing suffixes^66^.

#### Multilingual Naming Test

Traditionally, Multilingual Naming Test (MINT) is a language exam in which participants are shown several pictures and asked to name them. This test was modified from the original MINT and was made into a verbal naming exam for our study. The study coordinator read several descriptions of objects, landmarks, or time measurements, and the participant was instructed to name what was described. If the participant did not recall the object’s name on their initial try, a phonemic cue was given to assist with the word retrieval. A point was awarded for each correct item named. No points were awarded for correct names stated after a cue was given. The total number of points awarded for this task was 50^67^.

#### Wechsler Test of Adult Reading

The Wechsler Test of Adult Reading (WTAR) was first developed in 2001 to test an individual’s intelligence before an injury. In this task, the participant is presented with a list of 50 words divided into two separate columns. The participant is then instructed to read the list out loud to the research coordinator, starting with the first column, and then proceeding to the second column. The word list contains several peculiarities. All the words have some degree of unusual pronunciation that requires the participant to use their prior knowledge of the rules regarding word pronunciation, and not whether they are familiar with the word^68^.

#### Perceptual Speed

This is a paper-based attention and processing speed task where the participant is given two sheets of paper, each with 25 rows containing numbers one through nine placed randomly throughout each row and one number at the beginning of the row in parentheses. The objective is to scan each row visually for the number in parentheses at the left of the row and then cross it out with a pencil once it is seen. The goal is to maximize speed and accuracy within 2.5 minutes by completing as many rows as possible with the least number of errors/omissions. The results are graded on a combination of the number of correct cross-outs, incorrect cross-outs, and omitted choices^69^.

### Pre-processing and quantification of neuroimaging data

#### Reconstruction of T1-weighted structural MPRAGE scans

We used the FreeSurfer v.7 software package (http://surfer.nmr.mgh.harvard.edu/) to reconstruct each participant’s structural T1 scan. It segments cortical/subcortical areas based on each participant’s gyri and sulci morphology. White matter, gray matter, and cerebrospinal fluid boundaries were examined visually, slice by slice, by a single experienced technician for each participant, and if any visible discrepancy was detected, control points were added manually, repeating the reconstruction until the results became satisfactory^70^. Another expert technician performed second-level quality control, overlaying the borders of the segmented cortical/subcortical areas on top of the original image and eliminating inaccuracies in the segmentation process.

#### Pre-processing of task-based fMRI

An in-house preprocessing pipeline was implemented to ensure data quality and address specific challenges associated with multiband-planar imaging (EPI) sequences. All pre-processing steps are intentionally kept in the participants’ fMRI native space. This prevents any inaccuracy in spatial normalisation due to extensive atrophy in the elderly groups.

**Fig. 5:**
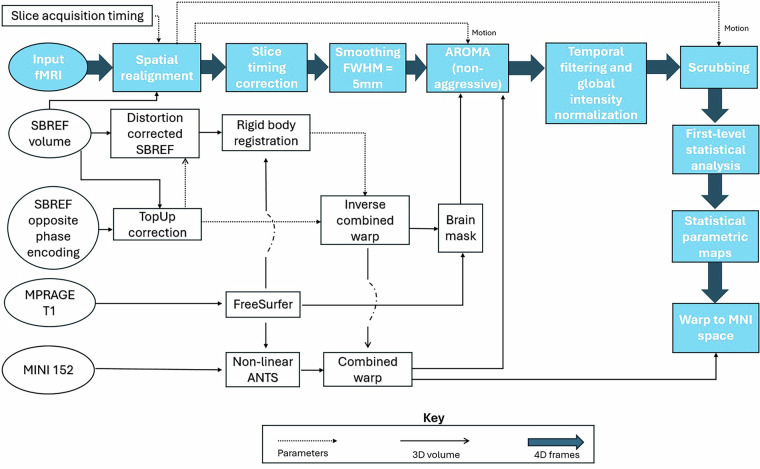
This figure illustrates the MRI processing pipeline for this dataset.

The flowchart of our pipeline is depicted in Fig. 5. It begins with spatial realignment, which was performed using FMRIB’s Linear Image Registration Tool Motion Correction (MCFLIRT) algorithm and a Single-Band Reference (SBRef) scan as the reference volume for the rigid-body registration to ensure accurate alignment of functional volumes through the course of the scan. Next, slice-acquisition-timing correction (temporal alignment) is performed using FSL (SLICETIMER), ensuring that all voxels’ time series are sampled at the beginning of the TR. Spatial smoothing was applied using FSL’s Smallest Univalue Segment Assimilating Nucleus (SUSAN) tool with a five mm Full-Width at Half-Maximum (FWHM) kernel to improve the signal-to-noise ratio while preserving spatial specificity.

To further remove motion-related artifacts, FSL’s ICA-based Automatic Removal of Motion Artifacts (AROMA) was employed to identify and remove the motion artifacts. The motion parameters obtained during the spatial realignment were fed to AROMA to help identify the motion-related independent components. AROMA also required an accurate brain mask and a warping field to transfer the participant’s fMRI native space to the Montreal Neurological Institute (MNI) template. The participant’s fMRI space, brain mask was generated using the participant’s structural scan (MPRAGE) and its FreeSurfer segmentation/parcellation. This FreeSurfer space brain mask, then inversely transformed to the participant’s fMRI space using the inverse of the rigid-body transformation and the inverse of the geometric distortion correction warping field obtained with FSL’s *TOPUP* tool, which we used to perform geometric distortion correction. The fMRI scan acquired before or after the current fMRI scan with opposite phase encoding direction was also fed to the TOPUP tool to estimate the warping field. To obtain a single warping field that transfers fMRI space to the MNI template, we used the Asymmetric Normalization Tools (ANTS) to perform a non-linear registration between the participant’s structural brain scans and the MNI152 brain template. The obtained warping field was then combined with the inverse of the geometric distortion correction warping field and the rigid-body transformation to give the single warping field that transforms the fMRI participant space scan into an MNI template. AROMA uses the motion parameters, fMRI space brain mask, and the combined warping field to identify motion-related artifacts and remove them from the fMRI scans. A non-aggressive AROMA was utilized since tb-fMRI scans are more robust to motion artifacts.

After AROMA, Global Intensity Normalization was performed by making sure the global median of the fMRI data intensity (after removing outliers) was 10000. Temporal Filtering was performed next with a high-pass band of f > 0.01 Hz. To remove all the remaining artifacts of the involuntary head motions, we performed scrubbing. Scrubbing was implemented to identify and exclude volumes with excessive motion (Framewise Displacement > 0.5 mm, or global gray matter percent change signal of root mean square deviation > 0.75). While the results of pre-processed fMRI data were in the participant’s native space, the combined warping field was used to warp both scrubbed and un-scrubbed pre-processed fMRI data into MNI space for future use in the higher-level analysis.

### Statistical analysis

First-level statistical analyses were conducted using FMRIB’s Improved Linear Model (FILM). The general linear model (GLM) was applied to the preprocessed data to identify task-related neural activity. Design matrices included task regressors convolved with the canonical double-gamma hemodynamic response function. Confounders included motion parameters and scrubbed volumes. Contrasts of interest were defined to identify the PBR and NBR^30^. The results of the first-level statistical analysis were kept in the participant’s fMRI native space. However, the combined warping field transformed the parametric statistical maps into MNI space for group-level analysis.

Second-level (group-level) analyses combined the MNI space statistical maps across participants using mixed-effects modelling, FMRIB’s Local Analysis of Mixed Effects (FLAME) stages 1 and 2. The parametric maps were fed into a group-level analysis to obtain group activation or contrast maps. Group-level modelling FLAME as part of the fMRI Expert Analysis Tool (FEAT) module for group-level analysis was used to obtain the single group-level average contrast maps. The parameter maps (voxel-wise regression coefficients and their variance) were sent to a second-level analysis to obtain the group map of the activations and deactivations using mixed-effects modeling. Single group-level averaging was used to get the group-level activation maps for each fMRI cognitive task. Statistical thresholding was performed using cluster-based correction for multiple comparisons, with a cluster-forming threshold of Z > 2.3 and a corrected cluster significance threshold of p < 0.05.

### Functional Connectivity analysis of fMRI

The same pre-processing pipeline as described for tb-fMRI data was also used for FC analysis. The only differences were 1) no smoothing was applied, 2) an aggressive AROMA was utilized instead of a non-aggressive AROMA, and 3) a low band-pass filter (0.01 < f < 0.1 Hz) was used instead of a high-pass filter. FreeSurfer segmentation/parcellation was inversely warped into the fMRI native space and used to extract WM and ventricular signals, which, along with motion parameters, were regressed out from all voxels’ time series. Next, FreeSurfer regional masks were used to extract regional time series. Other brain atlases, such as Schaefer, Power, and Coco, were also used to extract different sets of regional time-series and brain networks. These MNI space atlases were also inversely warped into the fMRI native space using the combined warping field explained in the fMRI pre-processing section. The conjunction of the native space gray-matter mask and the inversely warped regional masks were used to extract each atlas’s regional time series. Inter-regional FC was then computed using the Pearson correlation coefficient between regional time series. Network-wise FC was calculated by averaging all the pair-wise connections within each network.

### Quantification process for PET Data

We have developed an in-house, fully automatic quantification method^71^ and evaluated using histopathological data^72^, which has already been used in multiple studies on PET scans quantification^73–76^. Firstly, it aligns dynamic PET frames to the first frame using rigid-body registration, then averages them to generate a static PET image. It registers a structural T1 image in FreeSurfer space to the same participant’s static image using normalized mutual information and six degrees of freedom. This generates a rigid-body transformation matrix to transfer all FreeSurfer regional masks to static PET image space. It extracts the regional PET data by using these regional masks in the static PET space. It calculates the standardized uptake value (SUV) at selected regions and then normalizes it to cerebellum gray matter to derive the standardized uptake value ratio (SUVR). In the volumetric analysis of the FBB scan, the spill-in signal from nonspecific binding in white-matter is attenuated by discarding the uptake in the gray-matter voxels immediately adjacent to the white-matter volume, both in the cerebellum for computing the reference region uptake and in the cerebral cortex for obtaining cortical regions’ SUVR. For MK-6240, the non-specific binding is mainly in the meninges, so the same approach is used to discard all voxels on the convex hull of the brain’s surface.

#### De-identification, dataset, and sharing

Each participant who passed the initial screening and made their first appointment was assigned a participant ID number. This ID eliminated the need for identifying information (name, birth date, social security number) in upcoming data gatherings (clinical assessments, questionnaires, psychometric data, or neuroimaging data).

Original neuroimaging data were saved on the Quantitative Neuroimaging Lab (QNL) Digital Imaging and Communications in Medicine (DICOM) server. All neuroimaging data were anonymized with the assigned ID, converted to Neuroimaging Informatics Technology Initiative (NIfTI) format, and saved on three petabytes of QNL storage.

## Data Records

The dataset is currently available at the OpenNeuro (reference number: ds006148, https://openneuro.org/datasets/ds006148/versions, 10.18112/openneuro.ds006148.v1.0.5)^77^. We uploaded anonymous imaging and cognitive data in the Brain Imaging Data Structure (BIDS) format, where it is publicly available for research; however, to protect the privacy and identity of our participants, the demographics (age in years), sex, years of education, and race/ethnicity) are only available as a controlled access dataset. The name of this dataset is “The Multimodal Dataset to Investigate Task-Evoked Negative BOLD Response and Neurodegeneration”. Each participant is separated using a subject ID with a similar format (e.g., sub-C00321), and they may have up to three subfolders (i.e., ses-S001, ses-S002, and ses-S003) corresponding to sessions one, two, and three. Each session has subfolders titled as: “anat” (structural MRI), “func” (fMRI), “perf” (ASL images), “pet” (PET scans), and dwi (dMRI images). The images are in NIfTI format, and each has technical information in “.json” format. To gain access to the demographic dataset, researchers are instructed to download the Data Usage Agreement (DUA) under the “file-display” directory of the dataset (https://openneuro.org/datasets/ds006148/versions/1.0.5/file-display/DUA.pdf)^77^, fill it in and have it signed by their institution’s authorities and submit it for approval from the WCM to this email address, qrr4001@med.cornell.edu.

## Data Overview

Currently, 356 participants consented to the study (97 young aged 20–40 and 259 elderly aged 60–80; 182 males and 174 females). Of the 356 consented, 259 completed at least one scan. Of the 259 recruited participants, the young group comprised 62 individuals, with a mean ± SD age of 27.8 ± 5.3 years. The elderly group included 197 individuals, with a mean ± SD age of 68.6 ± 5.7 years. The average education level in the young group was 16.5 ± 2.0 years, while in the elder group, it was 17.0 ± 2.4 years. Of the total study population, 49% were female.

Most participants in both groups were White, with 31 young participants (50%) and 167 elderly participants (85%). Other ethnicities represented included African Americans (seven young, 15 elderly), Asian Americans/Pacific Islanders (twelve young, eight elderly), Hispanics (nine young, two elderly), and others (six young, eleven elderly).

This demographic data is detailed in Table 1, which outlines the breakdown of participants who completed at least one scan.

The scans resulted in 4688 MRI/fMRI scans (See Table 2) and 719 PET scans with three different tracers (See Table 3). Tables 2, 3 provide the details of the number of scans in each modality. In summary, 198 participants had all MRI/fMRI scans, and 224 participants (179 elderly and 45 young) had all three PET modalities. Finally, 189 participants had all the imaging scans. The number of excluded scans from MRI and PET studies is also included in Tables 2, 3, respectively. Scans acquired from one participant were excluded completely due to a large cystic lesion.

**Table 2:** MRI modality types completed by our cohort, separated by age group.

MRI Modality	Included	Young	Elderly	Excluded
Consented	356	97	259	N/A
MPRAGE	258	62	196	1
FLAIR	255	62	193	1
dMRI	243	57	186	1
pCASL	252	61	191	1
T2*	252	61	191	1
QSM/SWI	238	51	187	1
All MPRAGE & fMRI scans	198	48	150	N/A

**Table 3:** PET scan types completed by our cohort, separated by age group.

PET Modality	Included	Young	Elderly	Excluded
FDG PET	251	59	192	7
FBB PET	232	48	184	1
MK-6240 PET	236	52	184	1
Aβ, Tau, & FDG	224	45	179	1

### FDG PET Scans

In total, 251 FDG PET scans (192 elderly and 59 young) have been uploaded. In total, 7 FDG PET scans were excluded: 1 due to the cystic lesion and 6 due to missing T1-weighted structural scans. Table 4 summarizes the demographics of the 251 participants with FDG PET. We divided the participants into two groups, young and elderly, and listed their mean and standard deviation SUVR in each group.

**Table 4:** Summary of the demographics for 251 included participants for FDG PET imaging.

Group	Number	Sex Female (%)	Mean Age ± SD	Mean Global FDG SUVR ± SD
Young	59	29 (49.1%)	27.7 (±5.2)	1.16 (±0.07)
Elderly	192	96 (50%)	68.6 (±5.7)	1.07 (±0.07)

### FBB PET Scans

In total, 232 FBB scans (184 elderly and 48 young) have been uploaded. Table 5 lists the demographic information for the 232 participants, with included FBB PET. We divided the participants into three groups: young, Aβ−, and Aβ+, using the visual reading explained previously in the methods section. We identified 44 Aβ+ but cognitively unimpaired participants, whereas 140 participants were categorized as Aβ−, which makes the prevalence of Aβ positivity in elders at about 24%.

**Table 5:** Summary of the demographic data for 232 included participants for FBB PET imaging.

Group	Number	Sex Female (%)	Mean Age ± SD	Mean Global FBB SUVR ± SD
Young	48	24 (50.0%)	28.1 (±5.3)	1.08 (±0.02)
Aβ−	140	68 (48.6%)	67.6 (±5.5)	1.12 (±0.06)
Aβ+	44	23 (52.2%)	71.4 (±5.4)	1.50 (±0.30)

### MK-6240 PET Scans

In total, 236 MK-6240 scans (184 elderly and 52 young) have been uploaded. Table 6 summarizes the demographic data of 236 participants with included MK-6240 PET imaging. We divided the participants into three groups: young, Tau-, and Tau + , using the visual reading explained previously in the methods section. We identified 63 Tau + participants among our cognitively unimpaired elders, whereas 121 elders were categorized as Tau-, making the prevalence about 34%.

**Table 6:** Summary of the demographic data of 236 participants who completed MK-6240 PET imaging.

Group	Number	Sex Female (%)	Mean Age ± (SD)	Mean Global MK-6240 SUVR ± SD
Young	52	27 (51.9%)	28.2 ± 5.3	0.97 (±0.12)
Tau−	121	59 (48.8%)	67.3 ± 5.3	0.95 (±0.08)
Tau+	63	30 (47.6%)	71.3 ± 5.7	1.02 (±0.16)

### Functional connectivity scans (rs-fMRI)

Table 7 reports the number of rs-fMRI in our dataset. As shown, 34 participants (two young and 32 elderly) had two rs-fMRI scans each acquired in separate sessions, resulting in 4 young rs-fMRI and 64 elderly rs-fMRI scans. 219 participants (59 young and 160 elderly) had only one rs-fMRI scan. The total number of rs-fMRI scans uploaded to our dataset was 287, and 253 participants had either a single or double scan. We also excluded two rs-fMRI scans from our dataset due to unfinished scans.

**Table 7:** Number of uploaded rs-fMRI scans by age group and quantity (single or double).

Quantity	Total Number	Young	Elderly
Single rs-fMRI	219	59	160
Double rs-fMRI	68	4	64

#### Task-based fMRI Scans (tb-fMRI)

The number of scans that we included in each of the 12 tb-fMRI tasks and their age group are listed in Table 8. In summary, 258 participants have one or more scans, and 200 (49 young and 151 elderly) have all tasks. There are a total of 2903 tb-fMRI scans in our dataset. In addition to a complete exclusion of 12 tb-fMRI scans of one participant with a cystic lesion, we excluded 20 tb-fMRI scans due to unfinished tasks, E-Prime failure, or stimuli not properly presented.

**Table 8:** The total number of uploaded and excluded task-based fMRIs by cognitive domain.

Cognitive Domain	tb-fMRI Task	Included	Young	Elderly	Excluded
**Crystallized Memory**					
	Antonym	248	57	191	1
	Synonym	238	53	185	1
	Picture Vocabulary	236	53	183	3
**Episodic Memory**					
	Paired Associate	238	53	185	2
	Word Memorization	251	59	192	1
	Logical Memory	240	53	187	1
**Fluid Reasoning**					
	Matrix Reasoning	249	60	189	4
	Paper Folding	245	56	189	2
	Letter Sets	252	60	192	2
**Processing Speed**					
	Digit Symbol	234	57	177	8
	Pattern Comparison	244	58	186	5
	Letter Comparison	228	53	175	2

### APOE4 assay

The available data on the absolute and relative frequency of 131 participants with genetic testing is provided in Table 9. We have included 102 APOE4 negative participants and 29 positive participants. 26 participants were heterozygous, while three were homozygous.

**Table 9:** The total number of available APOE4 status.

	Absolute Frequency	Relative Frequency
Total Participants with Available Genetic Data	131	100%
APOE4 Negative	102	77.9%
APOE4 Positive	29	22.1%
Heterozygous Participants	26	19.8%
Homozygous Participants	3	2.3%

### Neuropsychological assessments

Table 10 lists the number of participants who completed each assessment battery and the numbers for the young and elderly groups. Out of the 356 participants who consented to the study, 216 completed all the neuropsychological tests, and 238 had at least some results. Of those who completed all tasks, 46 were young, and 170 were elderly. A total of 3973 neuropsychological assessments were completed for the whole cohort.

**Table 10:** The total number of outside scanner neuropsychological tasks uploaded by age group.

Outside Scanner Task	Total Number	Young	Elderly
Benson Complex Figure	235	51	184
Perceptual Speed	234	51	183
DKEFS	234	51	183
WTAR	236	51	185
Craft story	234	50	184
R-AVLT	228	49	179
Forward/Backward	232	49	183
Category Fluency	234	49	185
Verbal Fluency	235	50	185
Manikin	233	50	183
Shipley	235	50	185
Tower of London	233	51	182
Symbol Search	236	50	186
Trail Making	233	51	182
Four Choice Reaction Time	231	49	182
Verbal Naming	235	50	185
Baddeley	235	50	185

## Technical Validation

### FDG PET

**Fig. 6:**
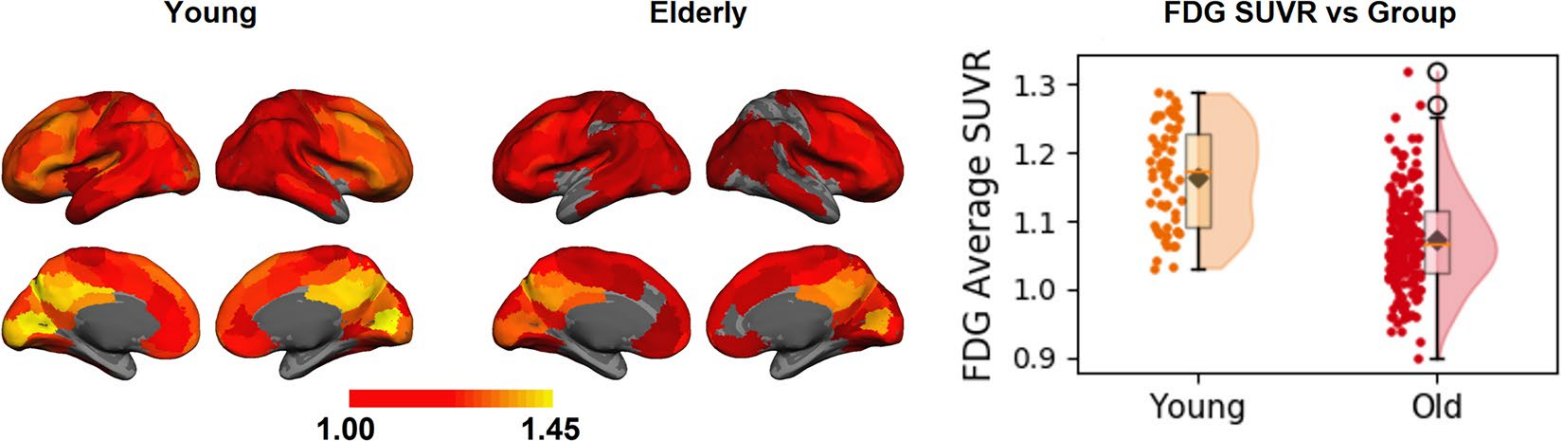
Mean regional (Schaefer 200) FDG SUVR is color-coded, thresholded (SUVR > 1), and projected on the surface of a semi-inflated cortex for young (left panel) and elderly (middle panel) participant groups separately. As expected, FDG SUVR is significantly lower in elderly participants. The right panel illustrates the raincloud plot of the FDG global SUVR, which shows the younger group had significantly (t = 8.58, p < 0.001) higher glucose consumption than the elderly group.

All FDG PET scans were processed and quantified using the approach explained in the methods section. The quantified FDG scans were averaged within each group, and their regional mean cortical FDG uptake was color-coded and projected on the semi-inflated surface of the cortex. (Fig. 6) The young group has higher regional glucose consumption than the elderly group. (Young group mean SUVR: 1.16, elderly group mean SUVR: 1.07) (Table 4) As a fast quality control, we performed a two-sample t-test between the young and elderly groups, which showed younger group as average had significantly (t = 8.58, p < 0.001) higher glucose consumption than that of the elderly group.

### FBB PET

**Fig. 7:**
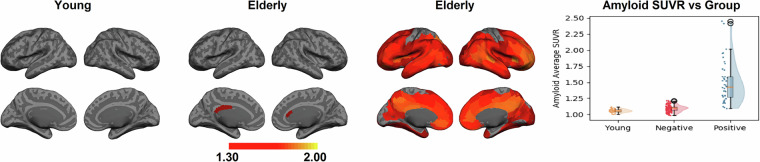
Voxel-wise mean Aβ SUVR are color-coded, thresholded (SUVR > 1.3), and projected on the surface of the semi-inflated cortex separately for the healthy young group (leftmost), Aβ− group (middle left), and Aβ+ group (middle right panel). As expected, Aβ SUVR is much higher in Aβ+ compared to Aβ− and young groups. Aβ negativity and positivity are based on visual reading. Finally, the distribution of the global Aβ SUVR for healthy young, Aβ−, and Aβ+ groups is shown with three raincloud plots in the rightmost last panel. One-way ANOVA across groups showed a significant difference (F = 137.99, p < 0.001).

Young participant data were used to generate the normative sample distribution for future statistical comparison and testing. The Aβ positivity rate was 23.9% in our elderly cohort. (Table 5) This is comparable to the existing reports^78–82^. All FBB PET scans were processed and quantified using the approach explained in the methods section. The quantified Aβ scans were averaged within each of the three groups: 1) Young , 2) Aβ−, and 3) Aβ+ participants. Figure 7 shows their voxel-wise mean SUVR color-coded and projected on the semi-inflated surface of the cortex. The subject-wise distribution of the global Aβ SUVR for young (mean = 1.08), Aβ− (mean = 1.12), and Aβ+ (mean = 1.5) is shown in the right panel (Fig. 7). As expected, Aβ SUVR is significantly higher in Aβ+ participants compared to Aβ− and young groups. One-way ANOVA across groups showed a significant difference (F = 137.99, p < 0.001), highlighting the technical validity of the FBB PET scans.

### MK-6240 PET

**Fig. 8:**
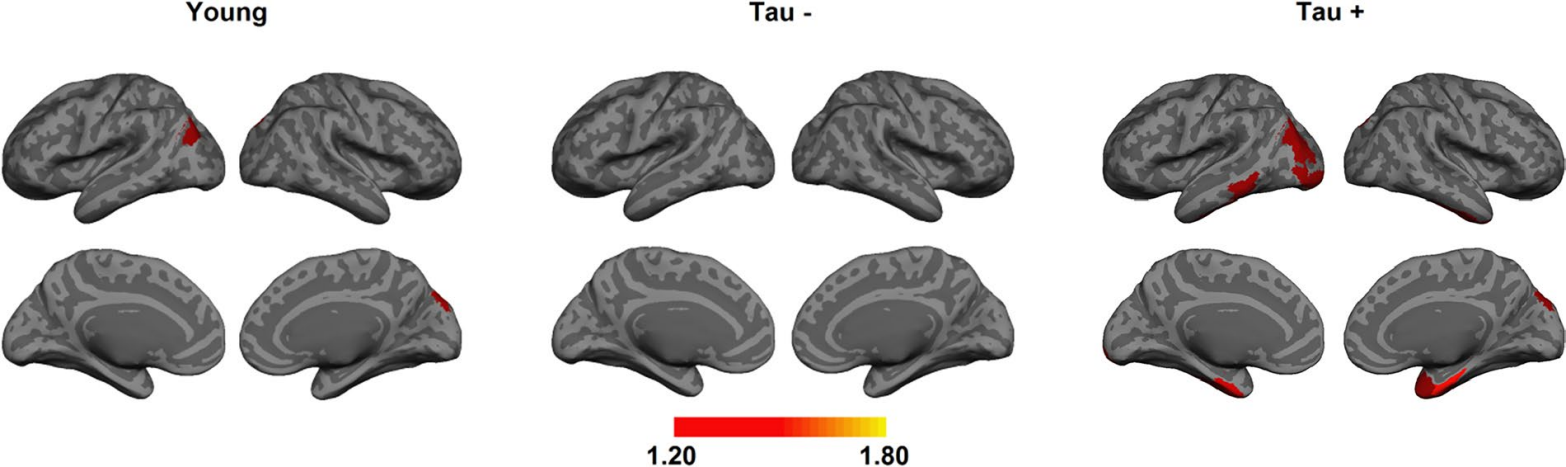
A comparison of Tau SUVR in three different regional surface plots each corresponding to a different group (bottom panel). The left panel is the young group, the middle panel is the Tau - group, and the right panel is the Tau + group. As expected, the Tau burden is substantially more prominent in the Tau + compared to Tau - and young individuals. Negativity and positivity are based on visual reading.

Young participants’ data were used to generate the normative sample distribution for future statistical comparison and testing. The positivity prevalence of tau deposition in our elderly cohort was approximately 34.2%. (Table 6) This is higher than the rates reported in the literature^79,82–84^. This may be due to our careful attention to the Medial Temporal Lobe (MTL) during visual reading and the higher specificity of the MK-6240 tau tracer in the MTL region than the Flortaucipir tracer^79^. This might be due to using the second-generation Tau tracer and more accurate visual reading. All MK-6240 PET scans were processed and quantified using the approach explained in the methods section. The quantified tau scans were averaged within each group: 1) Young, 2) Tau-, and 3) Tau+, and their voxel-wise SUVR were color-coded and projected on the semi-inflated surface of the cortex, as seen in Fig. 8. Figure 8 shows that young participants have a minor uptake in the visual cortex (Fig. 8, left panel). As expected, the Tau + group has a substantial uptake both in the MTL region and cortical regions outside the MTL (Fig. 8, right panel).

### Functional connectivity (rs-fMRI)

**Fig. 9:**
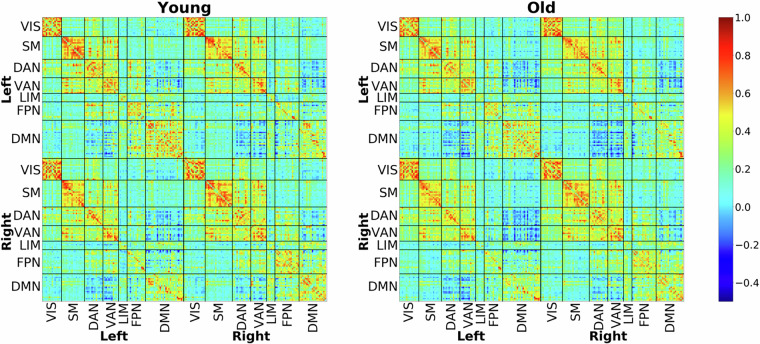
Inter-regional FC of the 200 Schaefer regions and 7 networks (Visual, Somatomotor, Ventral Attention, Dorsal Attention, Limbic, Frontoparietal, and Default Mode). The mean FC of the young and elderly groups was color-coded and depicted in separate cross-correlograms.

Using 200 regions from the Schaefer atlas, inter-regional Pearson Correlation Coefficients were obtained to determine the FC between all brain regions. Figure 9 shows the color-coded inter-regional connectivity on a cross-correlogram, where the regions of each network are clustered to highlight the difference within network connectivity versus across network connectivity. As shown in Fig. 9, mean FC correlation matrices were computed for every pairwise node from the Schaefer Atlas and depicted separately for young and elderly groups. As can be observed, the quality of our FC data is evident by showing the emergence of the seven FC networks with significantly higher correlation versus the lower correlation between network connectivity. Also, the observed anti-correlation between task negative and task positive networks lends support to this dataset as being of high quality.

On the other hand, the FC between the two groups did not seem to change substantially. These results might seem contradictory to existing literature, as age-related attenuation of the within-network FC is reported numerously in the literature^85–95^. To rule out the possibility that these discrepancies are indicative of the lack of quality in our rs-fMRI data, we took the following steps to ensure our rs-fMRI data has the required quality. First and most important item is head motion, which we meticulously put all our effort to control for by running AROMA, Scrubbing, and residualizing nuisance variables. This level of scrutiny was not common in the processing of the rs-fMRI scan, which often results in false-positive findings^89,96–98^. In fact, when we repeated the analysis without careful corrections of the head motion, we also found age-related differences in the within-network FC. Furthermore, adding each level of motion correction stepwise attenuated the age-related differences, as we have explained in our recent submission^28^. Second, we also noticed that the head motion always inflated the inter-regional correlations, but it never increased the anti-correlation between DMN and other brain networks. Therefore, instead of relying only on the age-related differences as our quality metric, we also considered the difference between intra-network FC and inter-network FC as our quality control metric. It was clear that participants with zero or negligible motion had a high level of within-network FC and a strong anti-correlation in between-network FC. As the motion increased, this difference was vanishing, even though all correlations were increasing. Adding each step of our motion correction pipeline, as mentioned above, improved the difference between intra- and inter-network FC, suggesting their proper motion correction application. We should emphasize that we did not regress out the global fMRI signal, since it inflates anti-correlations between brain networks, as it has been reported previously. Third, excluding participants with head motion and repeating the analysis with participants who had zero or minimal motion also resulted in no age-related differences in within-network FC. Fourth, our pipeline includes an extra step for controlling brain atrophy in elderly participants. By transferring the subject-specific gray-matter mask to MNI space, we made sure that we are only averaging the voxels that belong to gray-matter in each region. We have shown previously that inclusion of CSF and white matter voxel changes the BOLD response significantly in elderly participants^99^. Finally, while initial studies reported age-related attenuation of the FC network, many recent studies also report the resilience in the DMN FC across aging^100–102^. We hope that sharing this dataset with the research community will help discern the pattern of preserved or disturbed FC across aging and/or neurodegenerative disease.

### Tb-fMRI scans

**Fig. 10:**
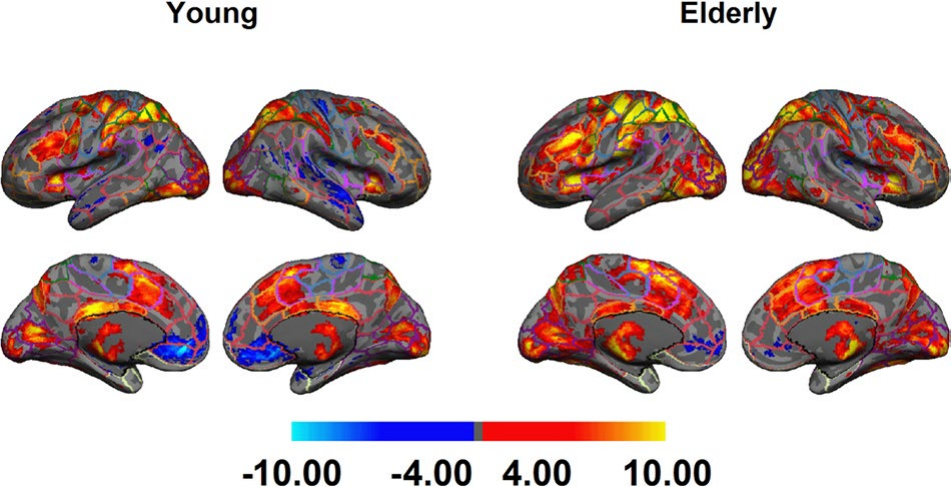
A Comparison of the young and elderly group’s mean NBR/PBR during Paired Associate tb-fMRI. These patterns are shown by projecting the color-coded voxel-wise significance of the activation/deactivation onto the semi-inflated cortex for young (Left panel) and elderly (Right Panel) groups. Activation of the bilateral visual cortex, as well as unilateral activation of the left sensory and motor cortex, and deactivation of the DMN, is noteworthy.

**Fig. 11:**
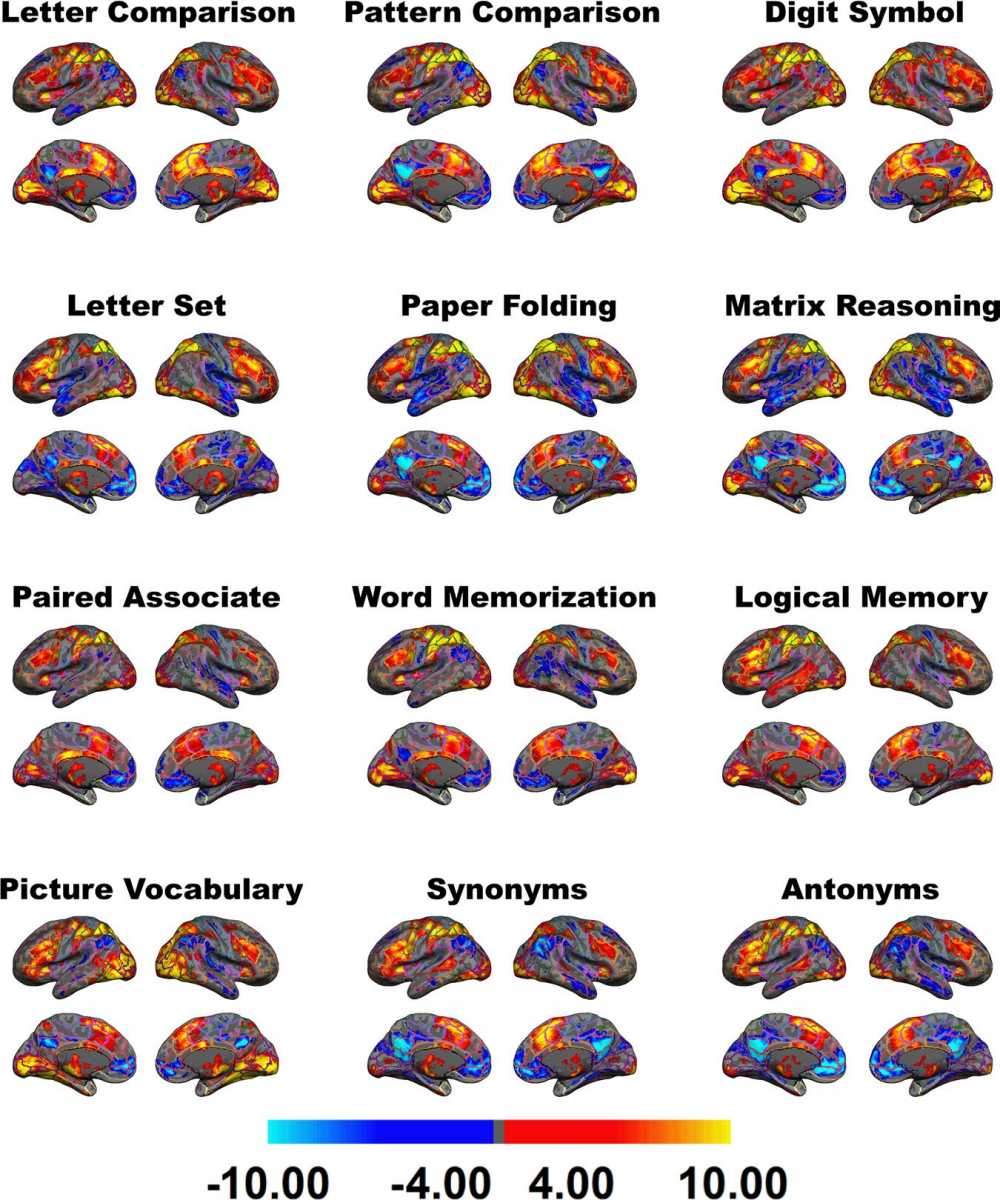
A Comparison of the young group’s mean NBR/PBR during twelve tb-fMRI scans. These patterns are shown by projecting the color-coded voxel-wise significance of the activation/deactivation onto the semi-inflated cortex for each task. Activation of the bilateral visual cortex, as well as unilateral activation of the left sensory and motor cortex, and deactivation of the DMN, is noteworthy.

To corroborate the technical validity of our dataset, the spatial pattern of brain activation/deactivation for the Paired Associate task is depicted in Fig. 10 by color-coding and projecting the significance of the activated/deactivated voxels (|z| > 4) onto the semi-inflated surface of the cortex. As shown, the Paired Associate task activates the bilateral visual cortex since the task is presented visually. There is also unilateral activation in the left sensory and motor cortices as they respond to the right hand (all participants are right-handed). Additionally, there are activations in the lateral frontal regions and the dorsal attention area. Finally, there are significant deactivations in the DMN regions (posterior cingulate, angular gyrus, medial-orbital-frontal, and middle temporal gyrus), especially in the young group. In addition, to demonstrate the quality of our tb-fMRI data in all tasks, we included the results of the group-level analysis (pattern of activation/deactivation) for young participants separately for each task. Examining the pattern of activation (visual cortex, left motor cortex, dorsal attention network (DAN), and executive control network) as well as the pattern of DMN deactivation, can be used as a measurement of quality in these tb-fMRI scans. The spatial pattern of young group brain activation/deactivation for all twelve tasks is depicted in Fig. 11.

**Fig. 12:**
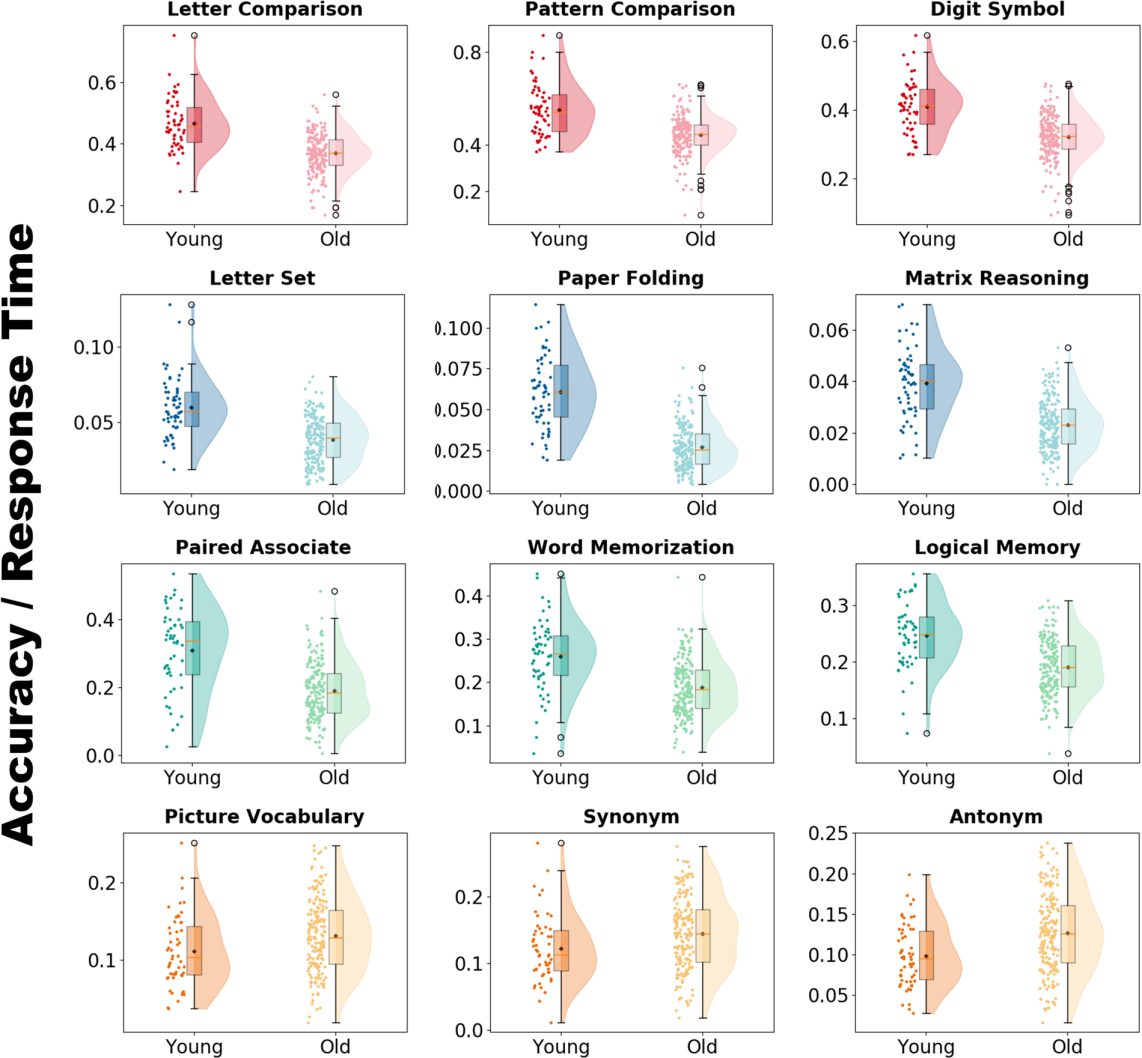
The distribution of the inside scanner performance depicted with separate rain-cloud plot for young and elders Increased ACC/RT corresponds to better performance. As expected, young participants performed better overall, as seen by the general trend of higher ACC/RT in most tasks. However, this was not the case for all three crystallized memory tasks, as their scores showed that elderly performed better.

In addition, within scanner task performance of the young and elderly groups is depicted in Fig. 12, using rain cloud plots (blue for young, red for elderly). Increased values of accuracy divided by response time (ACC/RT) correspond to better performance, assessed by performing a Student’s t-test between the group means of the two groups’ datasets. As expected, young participants performed much better overall, as seen by the general trend of higher ACC/RT in most tasks. However, this was not the case for all three crystallized memory tasks, as their scores indicated that elderly participants performed better in crystallized memory tasks. Similar results have been reported previously^103,104^. It is possible this may be due to the sampling of a healthier elderly cohort and slightly lower education in our young population, or to the accumulation of language ability over time within the elderly.

### APOE assay

Of the participants whose blood samples were analyzed, 22.1% were APOE 4 positive (2.3% of participants were APOE 4 homozygous). (Table 9) The results of our group’s analysis are congruent with those reported by others in community-based studies^105^.

### Neuropsychological assessments

**Fig. 13:**
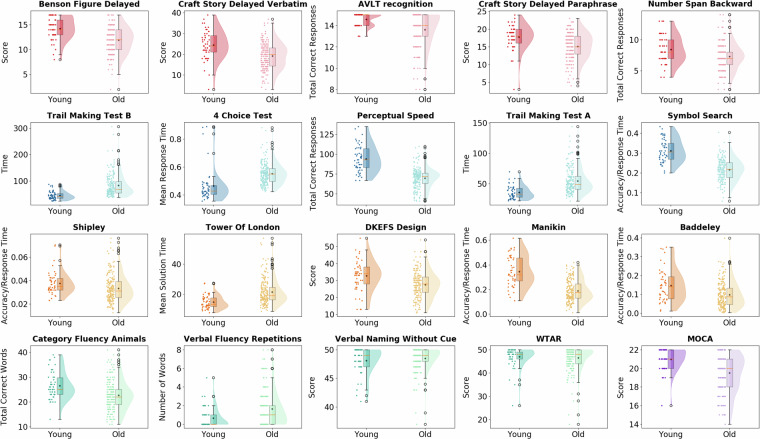
Distribution of the performance on the cognitive battery, and MoCA depicted separately for young and elderly participants using raincloud plots. As expected, young participants performed better.

In Fig. 13, we compare task performance between young and elderly participants using raincloud plots for outside scanner neuropsychological tasks. Decreased response time and increased scores correspond to better performance. As expected, young participants performed significantly better when their group mean differences were assessed using the Student t-test, which highlights the soundness of our dataset.

### Cognitive change index

The self-perceived change in cognitive abilities between young and elderly participants can be seen in Figs. 1, 2. Whereas young participants report no severe impairment in any of the nine cognitive abilities shown (~80% normal functioning, Fig. 1), there is a greater frequency of slight, mild, and moderate decline in all Cognitive Change Indexes and severe impairment in some abilities for the elderly group. (Fig. 2) This also confirms the validity of our data.

## Data Availability

The dataset is currently available at the OpenNeuro and is named QNL NegativeBOLD Dataset (reference number: ds006148, 10.18112/openneuro.ds006148.v1.0.5)^106^. Anonymized imaging and cognitive data can be downloaded in the BIDS format. Each participant is assigned a subject ID with a similar format (e.g., sub-C00321), and they may have up to three subfolders (i.e., ses-S001, ses-S002, and ses-S003) corresponding to sessions one, two, and three. Each session has subfolders titled as; “anat” (structural MRI), “func” (fMRI), “perf” (ASL images), “pet” (PET scans), and dwi (dMRI images). The images are in NIfTI format, and each has technical information in “.json” format^106^. Due to privacy concerns, the demographics are only available as a controlled-access dataset. To gain access to the demographic dataset, researchers are instructed to download the Data Usage Agreement (DUA) under the “file-display” directory of the dataset, fill it in and have it signed by their institution’s authorities and submit for approval from the WCM.

## Code availability

All custom codes are already shared on GitHub, and future development will be shared on our GitHub lab website (https://github.com/QuantitativeNeuroimagingLaboratory). The project’s main README file will also include instructions and parameter choices for the execution of these codes.
